# The Date of Interbreeding between Neandertals and Modern Humans

**DOI:** 10.1371/journal.pgen.1002947

**Published:** 2012-10-04

**Authors:** Sriram Sankararaman, Nick Patterson, Heng Li, Svante Pääbo, David Reich

**Affiliations:** 1 Department of Genetics, Harvard Medical School, Boston, Massachusetts, United States of America; 2 Broad Institute of MIT and Harvard, Cambridge, Massachusetts, United States of America; 3 Department of Evolutionary Genetics, Max Planck Institute for Evolutionary Anthropology, Leipzig, Germany; University of Washington, United States of America

## Abstract

Comparisons of DNA sequences between Neandertals and present-day humans have shown that Neandertals share more genetic variants with non-Africans than with Africans. This could be due to interbreeding between Neandertals and modern humans when the two groups met subsequent to the emergence of modern humans outside Africa. However, it could also be due to population structure that antedates the origin of Neandertal ancestors in Africa. We measure the extent of linkage disequilibrium (LD) in the genomes of present-day Europeans and find that the last gene flow from Neandertals (or their relatives) into Europeans likely occurred 37,000–86,000 years before the present (BP), and most likely 47,000–65,000 years ago. This supports the recent interbreeding hypothesis and suggests that interbreeding may have occurred when modern humans carrying Upper Paleolithic technologies encountered Neandertals as they expanded out of Africa.

## Author Summary

One of the key discoveries from the analysis of the Neandertal genome is that Neandertals share more genetic variants with non-Africans than with Africans. This observation is consistent with two hypotheses: interbreeding between Neandertals and modern humans after modern humans emerged out of Africa or population structure in the ancestors of Neandertals and modern humans. These hypotheses make different predictions about the date of last gene exchange between the ancestors of Neandertals and modern non-Africans. We estimate this date by measuring the extent of linkage disequilibrium (LD) in the genomes of present-day Europeans and find that the last gene flow from Neandertals into Europeans likely occurred 37,000–86,000 years before the present (BP), and most likely 47,000–65,000 years ago. This supports the recent interbreeding hypothesis and suggests that interbreeding occurred when modern humans carrying Upper Paleolithic technologies encountered Neandertals as they expanded out of Africa.

## Introduction

A much-debated question in human evolution is the relationship between modern humans and Neandertals. Modern humans appear in the African fossil record about 200,000 years ago. Neandertals appear in the European fossil record about 230,000 years ago [1] and disappear about 30,000 year ago. They lived in Europe and western Asia with a range that extended as far east as Siberia [2] and as far south as the middle East. The overlap of Neandertals and modern humans in space and time suggests the possibility of interbreeding. Evidence, both for [3] and against interbreeding [4], have been put forth based on the analysis of modern human DNA. Although mitochondrial DNA from multiple Neandertals has shown that Neandertals fall outside the range of modern human variation [5], [6], [7], [8], [9], [10], low-levels of gene flow cannot be excluded [10], [11], [12].

**Figure 1:**
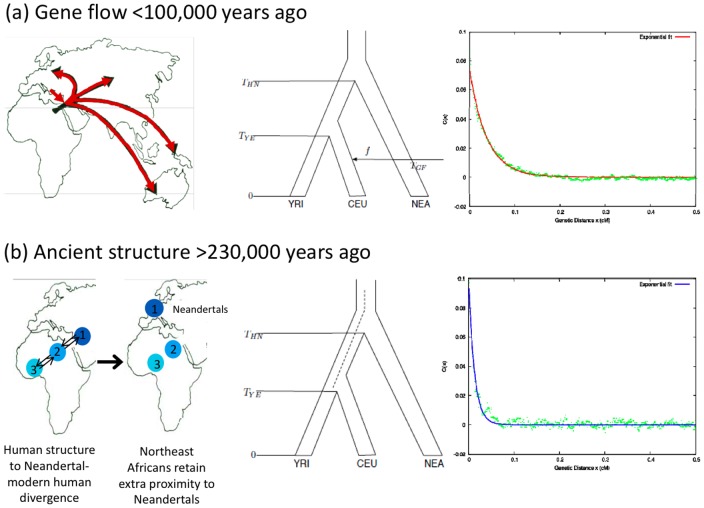
Linkage disequilibrium patterns expected due to recent gene flow and ancient structure. (A) In the case of recent gene flow from Neandertals (NEA) into the ancestors of non-Africans (CEU) but not into the ancestors of Africans (YRI), we expect long range LD at sites where Neandertal has the derived allele, and this expectation of admixture generated LD is verified by computer simulation as shown in the right of the panel along with a fitted exponential decay curve. (B) In the case of ancient structure, we expect short range LD, reflecting the time since Neandertals and non-Africans derived from a shared ancestral population, and this expectation is also verified by simulation.

Analysis of the draft sequence of the Neandertal genome revealed that the Neandertal genome shares more alleles with non-African than with sub-Saharan African genomes [13]. One hypothesis that could explain this observation is a history of gene flow from Neandertals into modern humans, presumably when they encountered each other in Europe and the Middle East [13] (Figure 1). An alternative hypothesis is that the findings are explained by ancient population structure in Africa [13], [14], [15], [16], whereby the population ancestral to Neandertal and modern human ancestors was subdivided. If this substructure persisted until modern humans carrying Upper Paleolithic technologies expanded out of Africa so that the modern human population that migrated was genetically closer to Neandertals, people outside Africa today would share more genetic variants with Neandertals that people in sub-Saharan Africa [13], [14], [15] (Figure 1). Ancient substructure in Africa is a plausible alternative to the hypothesis of recent gene flow. Today, sub-Saharan Africans harbor deep lineages that are consistent with a highly-structured ancestral population [17], [18], [19], [20], [21], [22], [23], [24], [25], [26], [27]. Evidence for ancient structure in Africa has also been offered based on the substantial diversity in neurocranial geometry amongst early modern humans [28]. Thus, it is important to test formally whether substructure could explain the genetic evidence for Neandertals being more closely related to non-Africans than to Africans.

A direct way to distinguish the hypothesis of recent gene flow from the hypothesis of ancient substructure is to infer the date for when the ancestors of Neandertals and a modern non-African population last exchanged genes. In the recent gene flow scenario, the date is not expected to be much older than 100,000 years ago, corresponding to the time of the earliest documented modern humans outside of Africa [29]. In the ancient substructure scenario, the date of last common ancestry is expected to be at least 230,000 years ago, since Neandertals must have separated from modern humans by that time based on the Neandertal fossil record of Europe [1].

In present-day human populations, the extent of LD between two single nucleotide polymorphisms (SNPs) shared with Neandertals can be the result of two phenomena. First, there is “non-admixture LD” [30] whose extent reflects stretches of DNA inherited from the ancestral population of Neandertals and modern humans as well as LD that has arisen due to bottlenecks and genetic drift in modern humans since they separated from Neandertals. Second, if gene flow from Neandertals into modern humans occurred, there is “admixture LD” [30], which will reflect stretches of genetic material inherited by modern humans through interbreeding with Neandertals. The extent of LD between single nucleotide polymorphisms (SNPs) shared with Neandertals will thus reflect, at least in part, the time since Neandertals or their ancestors and modern humans or their ancestors last exchanged genes with each other.

The strategy of using LD to estimate dates of gene flow events has been previously been explored by several groups [31], [32], [33], [34], [35]. Our methodology is conceptually similar to the methodology developed by Moorjani et al., but is dealing with a more challenging technical problem since the methodology developed by Moorjani et al. is adapted for relatively recent admixtures. In recently admixed populations that have not experienced recent bottlenecks, admixture LD extends over size scales at which non-admixture LD makes a negligible contribution. Thus, one can infer the time of gene flow based on inter-marker spacings that are larger than the scale of non-admixture LD. For older admixtures however (such as may have occurred in the case of Neandertals), non-admixture LD occurs almost at the same size scale as admixture LD. To account for this, we study pairs of markers that are very close to each other, but ascertain them in a way that greatly minimizes the signals of non-admixture LD while enhancing the signals of admixture LD. Thus, unlike in the case of recent admixtures, non-admixture LD could bias an admixture date obtained using our methods; however, we show using simulations of a very wide set of demographic scenarios that our marker ascertainment procedure makes the bias so small that our inferences are qualitatively unaffected.

Our methodology is based on the idea that if two alleles, a genetic distance *x* (expected number of crossover recombination events per meiosis) apart, arose on the Neandertal lineage and introgressed into modern humans at time *t_GF_*, the probability that these alleles have not been broken up by recombination since gene flow is proportional to 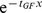. We show that the LD across introgressed pairs of alleles is expected to decay exponentially with genetic distance. The rate of decay is informative of the time of gene flow and is robust to demographic events (Appendix A, Text S1). In practice, we need to ascertain SNPs that, assuming recent gene flow occurred, are likely to have arisen on the Neandertal lineage and introgressed into modern humans. We choose a particular ascertainment scheme and show, using simulations of a number of demographic models, that the exponential decay of LD across pairs of ascertained SNPs provides accurate estimates of the time of gene flow. A second potential source of bias in estimating ancient dates arises from uncertainties in the genetic map. We develop a correction for this bias and show that this correction yields accurate dates in the presence of uncertainties in the genetic map. Combining these various strategies, we are able to obtain accurate estimates of the date of last exchange of genes between Neandertals and modern humans (also see Discussion). This date shows that recent gene flow between Neandertals and modern humans occurred but does not exclude that ancient substructure in Africa also contributes to the LD observed.

## Results

To study how LD decays with the distance in the genome, we computed the average value, 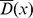
_,_ of the measure of linkage disequilibrium *D* (the excess rate of occurrence of derived alleles at two SNPs compared with the expectation if they were independent [36]) between pairs of SNPs binned by genetic distance *x* (see Methods). Immediately after the time of last gene flow between Neandertal (or their relatives) and human ancestors, long range LD is generated, and it is then expected to decay at a constant rate per generation as recombination breaks down the segments shared with Neandertals. Thus, in the absence of new LD-generating events (discussed further below), the 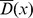 statistic across pairs of introgressed alleles is expected to have an exponential decay with genetic distance, and the genetic extent of the decay can thus be interpreted in terms of the time of last shared ancestry between Neandertals (or their relatives) and modern humans (Section S1 and Appendix A in Text S1).

To amplify the signal of admixture LD relative to non-admixture LD, we restricted our analysis to SNPs where the “derived” allele (the one that has arisen as a new mutation as determined by comparison to chimpanzee) is found in Neandertals and occurs in the tested population at a frequency of <10%. The justification for this frequency threshold is two-fold. First, the signal of Neandertals being more closely related to non-Africans than to Africans is substantially enriched at SNPs below this threshold (Section S1 in Text S1). Second, under the model of recent gene flow, such SNPs have an increased probability of having arisen due to mutations on the Neandertal lineage; we estimate that about 30% of them will have arisen on the Neandertal lineage under a model of history that we fitted to the data. This ascertainment enriches the class of informative SNPs by a factor of ten (Section S1 in Text S1). Our simulations show that restricting to this class of SNPs yields accurate estimates of the time of gene flow for a wide range of demographic histories consistent with patterns of human variation (Section S2 in Text S1).

**Figure 2:**
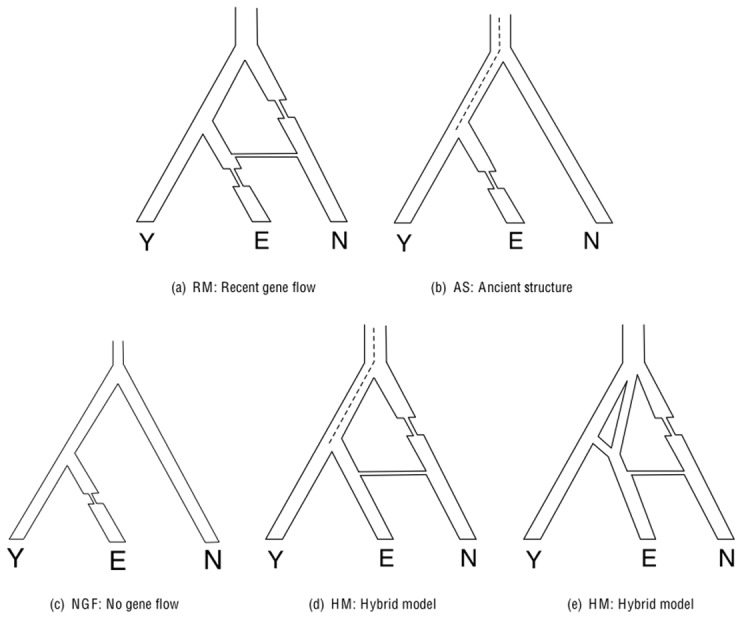
Classes of demographic models relating Africans (Y), Europeans (E), and Neandertals (N). a) Recent gene flow but no ancient structure. RGF I has no bottleneck in E. RGF II has a bottleneck in E after gene flow while RGF VI has a bottleneck in E before the gene flow. RGF IV and V have constant population sizes of N_e_ = 5000 and N_e_ = 50000 respectively. b) Ancient structure but no recent gene flow. AS I has a constant population size while AS II has a recent bottleneck in E. c) Neither ancient structure nor recent gene flow. NGF I has a constant population size while NGF II has a recent bottleneck in E. d),e) Ancient structure+Recent gene flow. HM IV consists of continuous migration in the Y-E ancestor and the Y-E-N ancestor while HM I consists of continuous migration only in the Y-E ancestor. HM II consist of a single admixture event in the ancestor of E while HM III also models a small population size in one of the admixing populations.

To assess how useful this statistic is for measuring admixture LD, we performed coalescent simulations of 100 regions of a million base pairs each, for a range of demographic histories chosen to be plausible for Neandertals, West Africans and non-Africans (these histories were constrained by the observed population differentiation between west Africans and Europeans as measured by their F_ST_ and the quantitative extent to which Neandertals share more derived alleles with Europeans than with Africans). The simulation results, which we discuss at length in Section S2 of Text S1, and summarize in Table 1, show that we obtain accurate and relatively unbiased estimates of the number of generations since admixture (never more than 15% from the true value) for (1) constant-sized population scenarios, (2) demographic models that include population bottlenecks as well as more recent admixture after the gene flow, (3) hybrid models of ancient structure and recent gene flow, and (4) mutation rates that differ by a factor of 5 from what we use in our main simulations ( see Figure 2). Two other SNP ascertainment schemes yield qualitatively consistent findings but the ascertainment we used provides the most accurate estimates under the range of demographic models considered (Section S5 of Text S1 and Table 1). The simulations also show that in the absence of gene flow (including in the scenario of ancient subdivision), the dates obtained are always at least 5,000 generations for scenarios of demographic history that match the constraints of real human data. Thus, an empirical estimate of a date much less than 5,000 generations likely reflects real gene flow.

**Table 1:** Estimates of the time of gene flow for different demographic models and mutation rates as well as different ascertainments.

Demography	Fst (Y,E)	D(Y,E,N)	Ascertainment 0	Ascertainment 1	Ascertainment 2
**No ancient structure and no gene flow**					
NGF I	0.15	0	8847±126	7940±257	10206±280
NGF II	0.15	0	5800±164	7204±356	11702±451
**Ancient structure**					
AS I	0.15	0.045	10128±127	8162±107	8861±110
AS II	0.19	0.046	5070±397	6349±327	7570±433
**Gene flow 2,000 generations ago**					
RGF II	0.15	0.041	1987±48	1693±39	1960±43
RGF III	0.14	0.043	1776±87	1643±98	2272±102
RGF IV	0.15	0.04	2023±56	1751±36	1995±38
RGF V	0.07	0.04	2157±22	2094±22	2105±22
RGF VI	0.15	0.04	2102±36	1814±35	2029±38
**Hybrid models of ancient structure and gene flow 2,000 generations ago**					
HM I	0.18	0.03	2174±40	2057±30	2228±38
HM II	0.12	0.04	2226±39	2049±30	2100±30
HM III	0.13	0.04	2137±34	2040±29	2124±30
HM IV	0.18	0.06	2153±36	2038±34	2187±35
**Gene flow 2,000 generations ago along with a varying mutation rate**					
μ = 1×10^−8^/bp/gen.	0.11	0.04	2141±41	1847±35	1969±36
μ = 5×10^−8^/bp/gen.	0.11	0.04	2134±41	1833±29	1951±29

The table presents estimates of the time of gene flow for different demographic models and mutation rates as well as different ascertainments. The main classes of models are a) NGF: No gene flow in a randomly mating population; b) AS: Ancient structure, c) RGF : Recent (2,000 generation ago) gene flow from Neandertals (N) into European ancestors (E), d) HM: Hybrid models with ancient structure and recent gene flow and e) Mutation rates that are set to 1×10^−8^/bp/generation and 5×10^−8^/bp/generation. The parameters of the models were chosen to match observed F_ST_ between Africans (Y) and Europeans (E) and to match the observed D-statistics of Africans and Europeans relative to Neandertal D(Y,E;N). In all models that involve recent gene flow, the time of gene flow was set to 2,000 generations. Our estimator of the time of gene flow provides accurate estimates of the time of gene flow for a wide range of demographic and mutational parameters. More details on the models and the ascertainments are in Figure 2, Figures S2 and S5 in Text S1.

We applied our statistic to data from Pilot 1 of the 1000 Genomes Project, which discovered polymorphisms in 59 West Africans, 60 European Americans, and 60 East Asians (Han Chinese and Japanese from Tokyo) [37]. We binned pairs of SNPs by the genetic distance between them using the deCODE genetic map. We considered all pairs of SNPs that are at most 1 cM apart. We computed the average LD over all pairs of SNPs in each bin and fit an exponential curve to the decay of LD (from 0.02–1 cM in 0.001 cM increments).

**Figure 3:**
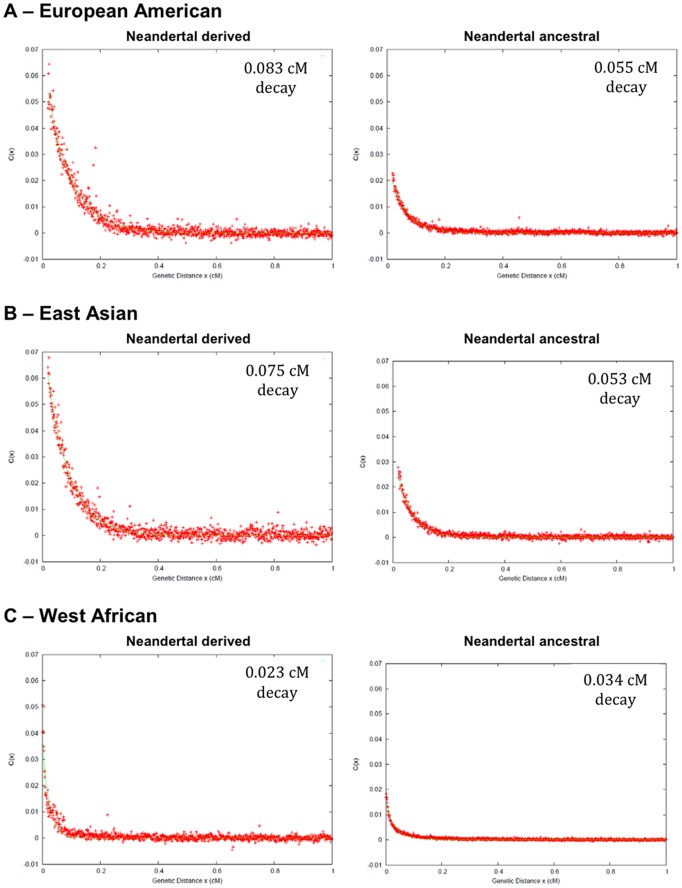
Decay of LD for SNPs with minor allele frequency <10%. (A, B) Real data for European Americans and East Asians shows longer range LD when the Neandertal genome has the derived allele (left) than when it has the ancestral allele (right). This is as expected due to gene flow from Neandertal, but is not expected in the absence of gene flow. In other words, the fact that LD conditional on Neandertals having the derived allele is longer than LD when Neandertal does not strongly suggests that the pattern we are observing among ascertained SNPs is reflecting the complex historical relationship between non-African modern humans and Neandertals, the signal we care about here, and not demographic events that solely involve the ancestors of non-Africans. The scale of the LD decay (1/*e* drop of the fitted exponential curve) is shown in the top right of each panel based on the deCODE genetic distance. (In Figure S8 of Text S1, we show that this signal persists when stratified into narrow allele frequency bins.) (C) In West Africans the pattern is qualitatively different such that when Neandertal is derived at both SNPs, LD decays more quickly than when Neandertal is ancestral at both SNPs, as expected in the absence of gene flow (without gene flow, the derived allele is always expected to be older so LD is expected to have had more time to break down).

Figure 3 shows the extent of LD for pairs of SNPs where both SNPs have a derived allele frequency <10%. This figure shows that the extent of LD is larger in Europeans and East Asians than in West Africans, both when the Neandertal genome carries the derived and when it carries the ancestral allele. Empirical features of these LD decay curves show that, for alleles derived in the Neandertal genome, the pattern in Europeans and East Asians is reflecting “admixture LD”. LD in West Africans is less extensive when Neandertals carry the derived allele than when they carry the ancestral allele, while the reverse is seen in Eurasians. To understand this, we note that in the absence of gene flow, polymorphic sites where Neandertals carry the derived allele must have arisen from mutations that occurred prior to Neandertal-human divergence so that they are old and recombination will have had a lot of time to break down the LD, while sites where Neandertals carry the ancestral allele mutations will include mutations that have arisen since the Neandertal-human split and thus LD will be expected to be more extensive, exactly as is seen in West Africans. In contrast, if gene flow occurred, then LD can be greater at sites where Neandertals carry the derived allele as is observed in Europeans and East Asians. This signal persists when we stratify the LD decay curves by the frequency of the ascertained SNPs (Figure S8 in Text S1). Thus the scale of the LD at these sites must be conveying information about the date of gene flow.

A concern in interpreting the extent of LD in terms of a date is that all available genetic maps (which specify the probability of recombination per generation between all pairs of SNPs) are likely to be inaccurate at the scale of tens of kilobases that is relevant to our analysis. We confirmed that errors in genetic maps can bias LD-based date estimates by simulating a gene flow event 2,000 generations ago using a model in which recombination was localized to hot spots [38] but where the data were analyzed assuming a genetic map that assumed homogeneous recombination rates across the genome. This led to a date of 1,597 generations since admixture. We developed a statistical model of the random errors that relate the true and observed genetic maps (see Methods). The precision of the map is modeled using a scalar parameter α. A unit interval of the observed genetic map corresponds to an interval in the true map of expected unit length and variance 1/α. To validate this error model, we estimated the map error in these simulations (α) by comparing the true and the observed genetic maps. Theoretical arguments (Section S3 in Text S1) show that we can obtain a corrected date (*t_GF_*) from the uncorrected date in generations (λ) using the equation *t_GF_* = α(e^λ/α^-1). We applied this correction to obtain a date of 1,926 generations. While this error model appears to provide an adequate description of random errors in a genetic map, it does not account for systematic biases.

To apply this statistical correction to real data, we estimated the error rate α in the genetic map by comparing the genomic distribution of a set of cross-over events from 728 meioses previously detected in a European American Hutterite pedigree [39] to what would be expected if the map were perfect. Unfortunately, the map that we would ideally want to use for estimating the date of Neandertal admixture is not the genetic map that applies to Hutterites today, but the time-averaged genetic map that applied between the present and the date of gene flow. Obviously, such a map is not available, but we hypothesize that by performing our analyses using a genetic map that is built from samples more closely related to the Hutterite pedigree than the map that we would like to analyze (the deCODE pedigree map built in Icelanders) as well as a genetic map that averages over too long a period of time (the European LD Map, which measures recombination over approximately five hundred thousand years), we can obtain some sense of the robustness of our inferences to uncertainties in how the European genetic map has changed over time.

Table 2 shows the estimates of λ, α and *t_GF_* in Europeans obtained using the two genetic maps. The estimates of *t_GF_* are in 1,805–2,043 over the deCODE and European LD maps. We also estimated λ in East Asians using the “East Asian LD map”. We find that λ in East Asians based on the East Asian LD map is 1,253–1,287, similar to the 1,159–1,183 in Europeans based on the European LD map, although the similarity of these numbers does not prove the Neandertal genetic material in Europeans and East Asians derives from the same ancestral gene flow event. While a shared ancestral gene flow event is plausible, the gene flow events could in principle have occurred in different places at around the same time [40]. We also cannot reliably estimate the recombination rate correction factor α for the East Asian map because we do not have access to cross-over events in an East Asian pedigree, and hence we do not present an estimate of *t_GF_* in East Asians and focus on Europeans in the rest of this paper.

**Table 2:** Admixture dates for Europeans.

Map	λ (95% credible interval)	*t_GF_* (generations) (95% credible interval)	y*_GF_* (years) (95% credible interval)
Decode	1,179–1,233	1,805–1,993	47,334–63,146
European LD	1,159–1,183	1,881–2,043	49,021–64,926

The table gives the admixture dates for Europeans. For East Asians we obtain λ = 1,253–1,287, although no valid conversion to *t_GF_* is possible without an East Asian pedigree map and hence we focus on the results for Europeans in this study.

To convert the date estimates in generations to date estimates in years, we use an average generation interval which has been estimated to be 29 in diverse modern hunter gatherer societies as well as in developing and industrialized nation states [41]. We assume a uniform prior probability distribution of generation times between 25 and 33 years per generation for the true value of this quantity and integrate this with the uncertainty of λ and α, and obtain an estimate of last gene exchange between Neandertals and European ancestors of 47,334–63,146 years for the deCODE map, and 49,021–64,926 years for the European LD Map (95% credible intervals). Taking the conservative union of these ranges, we obtain 47,000–65,000 years BP. In our simulations of ascertainment strategy, we found demographic models that can produce biases in the date estimates that could be as large as 15% (Section S2 in Text S1). To be conservative, we applied this to the uncorrected dates from each of the maps and then applied the relevant map correction. The union of the resulting intervals leads us to conclude that the true date of gene flow could be as young as 37,000 years BP or as old as 86,000 years BP.

We considered the possibility that our results might be biased by natural selection, which is known to affect patterns of human genetic diversity and to have had a much larger effect closer to genes [42], [43]. We estimated the time of gene flow stratifying the SNPs by their distance to the nearest exon, dividing the data into 5 bins such that each bin contained 20% of all the SNPs. Using the deCODE map, we obtain λ = 1,145–1,301 in all bins (Table S8 in Text S1). This estimate is concordant with the λ = 1,201 obtained without stratification, and suggests that our inferences are not an artifact of LD generated by directional natural selection.

## Discussion

The date of 37,000–86,000 years BP is too recent to be consistent with the “ancient African population structure” scenario, and strongly supports the hypothesis that at least some of the signal of Neandertals being more closely related to non-Africans than to Africans is due to recent gene flow. These results are concordant with a recent paper by Yang et al [44] that analyzed joint allele frequency spectra in Africans, non-Africans and Neandertals, to reject the ancient structure scenario. After the present paper was accepted, Eriksson and Manica [45] showed, using an Approximate Bayesian Computation approach, that models of ancient substructure can produce a signal of Neandertals sharing more derived alleles with non-Africans than with Africans (that is, they can account for the observation that D-statistics are significantly different from zero). The same observation was made in our earlier papers on the draft Neandertal and Denisovan genomes where we introduced D-statistics [13], [14]. However, the new statistics we focus on here as well as the statistics focused on by Yang et al [44] show that ancient structure alone cannot explain these signals.

One possibility that we have not ruled out is that both ancient structure and gene flow occurred in the history of non-Africans. In the simulations reported in Table 1, we show that in this scenario, the ancient structure will tend to make the date estimate older than the truth but by not more than 15%, so that the date of 37,000–86,000 should still provide a valid bound while the less conservative estimate of 47,000–65,000 years should be interpreted as an upper bound on the date of gene flow.

Further, we have not been able to differentiate amongst variants of the recent gene flow scenario: a single episode or multiple episodes of gene flow or continuous gene flow over an extended period of time. Our date has a clear interpretation as the time of last gene exchange under a scenario of a single instantaneous gene flow event. In the other scenarios, the date is expected to represent an average over the times of gene flow and should be interpreted as an upper bound on the time of last gene exchange.

While recent gene flow from Neandertals into the ancestors of modern non-Africans is a parsimonious model that is consistent with our results, our analysis cannot reject the possibility that gene flow did not involve Neandertals themselves, but instead populations that were more closely related to Neandertals than any extant populations are today. Thus, the date should be interpreted as the last period of time when genetic material from Neandertals or an archaic population related to Neandertals entered modern humans.

Genetic analyses by themselves offer no indication of where gene flow may have occurred geographically. However, the date in conjunction with the archaeological evidence suggests that the two populations likely met somewhere in Western Eurasia. An attractive hypothesis is the Middle East, where archaeological and fossil evidence indicate that modern humans appeared before 100,000 years ago (as reflected by the modern human remains in Skhul and Qafzeh caves), Neandertals expanded around 70,000 years ago (as reflected for example by the Neandertal remains at Tabun Cave), and modern humans re-appeared around 50,000 years ago [29]. Our genetic date estimates, which have a mostly likely range of 47,000–65,000 years ago (and are confidently below 86,000 years ago), are too recent to be consistent with the appearance of the first fossil evidence of modern humans outside of Africa—that is, our date makes it unlikely that the Neandertal genetic material in modern humans today could arise exclusively due to the gene flow involving the Skhul/Qafzeh modern humans—and instead point to gene flow in a more recent period, possibly when modern humans carrying Upper Paleolithic technologies expanded out of Africa.

## Methods

### Linkage disequilibrium statistic

Our procedure computes a statistic based on the LD observed between pairs of SNPs. For all pairs of ascertained SNPs at a genetic distance *x*, we compute the statistic:
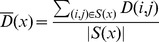
Here *S(x)* denotes the set of all pairs of ascertained SNPs that are at a genetic distance *x*, and *D(i,j)* denotes the classic signed measure of linkage disequilibrium, D, at the SNPs *i, j*. The sign of *D(i,j)* is determined by computing D using the derived alleles (defined relative to the chimpanzee base) at SNPs *i* and *j*. Under the gene flow scenario, we expect the contribution of introgression to 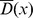 to have an exponential decay with rate equal to the time of gene flow, provided the gene flow is more recent than the Neandertal-modern human split (Section S1 and Appendix A of Text S1).

We pick SNPs that contain a derived allele in Neandertal (defined relative to the chimpanzee base) and are polymorphic in the target population with a derived allele frequency <10%. Further details can be found in Text S1, along with simulations exploring the performance of the statistic and demonstrating its properties under various demographic models and ascertainment schemes.

### Preparation of 1000 Genomes Data and alignment to chimpanzee and Neandertal

We used the 1000 Genomes Pilot 1 genotypes to estimate the LD decay. For each of the panels that were chosen as the target population in our analysis, we restricted our analysis to polymorphic SNPs. The SNPs were polarized relative to the chimpanzee base (*panTro2*).

### Computation of the LD statistic on 1000 Genomes Data

For the set of ascertained SNPs, we compute 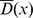 as a function of the genetic distance *x* and fit an exponential curve using ordinary least squares for *x* in the range of 0.02 cM to 1 cM in increments of 0.001 cM. The standard definition of *D* requires the availability of haplotypes. We instead computed *D(i,j)* as the covariance between the genotypes observed at SNPs *i* and *j* [46]. Simulations show that dates estimated using this definition of *D* on unphased genotypes are very similar to the estimates obtained from haplotypes (Section S2.1.1 of Text S1). We were concerned that the complicated method used in the 1000 Genomes Project for determining genotypes, which involved statistical imputation and probabilistic calling of genotypes based on LD, might in some way be biasing our inferences based on LD. Thus, we also computed *D(i,j)* for all pairs of SNPs that passed our basic filters (SNPs that contain a derived allele in Neandertal and are polymorphic in the target population with derived allele frequency <10% as estimated from the reads) by computing LD directly from the reads, again using the SAMtools package [47], and obtain qualitatively consistent results (Section S7 of Text S1). Further, simulations to mimic the low power to call rare SNPs in the 1000 genomes data show that our estimates are not sensitive to the deficit of rare alleles (Section S6 of Text S1).

### Correction for error in the genetic map

We have a genetic map G defined on *m* markers. Each of the *m-1* intervals is assigned a genetic distance *g_i_, i = 1,..m-1*. These genetic distances provide a prior distribution for the true underlying (unobserved) genetic distances *Z_i_*. A reasonable prior on each *Z_i_* is then:
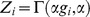
where α is a parameter that is specific to the map. This implies that the true genetic distance *Z_i_* has mean *g_i_* and variance *g_i_/*α. Thus, large values of α correspond to a more precise map. A motivation for the choice of the gamma prior over *Z_i_* is that this prior has the key invariance property *Z_1_+Z_2_*∼Γ(*α(g_1_+g_2_),α*). Thus, α is a property of the map and not of the specific markers used.

Given this prior on the true genetic distances, fitting an exponential function to pairs of markers at a given observed genetic distance *g* involves integrating over the exponential function evaluated at the true genetic distances given observed genetic distance *g*, that is:
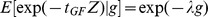
where λ is the rate of decay of 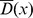 as a function of the observed genetic distance *g* and can be estimated from the data as described in the previous section, *t_GF_* denotes the true time of the gene flow and the expectation is over the unobserved true genetic distance *Z*. We can use this equation to solve for *t_GF_* as (see Appendix B in Text S1):
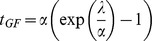
To estimate α for a given genetic map, we propose a statistical model that relates the true unobserved genetic map to the observed map and to crossover events found in a pedigree. We estimate the posterior distribution of α by Gibbs sampling (Section S3 of Text S1).

### Uncertainty in the date estimate taking into account all sources of error

To obtain estimates of the time of gene flow taking into account all sources of error, we formulated a Bayesian model that relates λ, *t_GF_*, and *y_GF_* (the time in years) (Section S4 of Text S1) to the observed LD decay curve.

Further, we assume a uniform prior distribution on the number of years per generation of 25–33 years, based on a recent survey of generation intervals, which are similar in diverse hunter-gatherer societies and in undeveloped as well as industrialized nation states.

Assuming a flat prior on each of λ, *t_GF_*, and *y_GF_* , we use Gibbs sampling to obtain samples from the posterior distributions of each of these parameters. We then report the posterior mean and 95% Bayesian credible intervals.

### Availability

We will make the data and programs available at http://genetics.med.harvard.edu/reichlab/Reich_Lab/Datasets.html on publication.

## Supporting Information

Text S1 Supporting Text including Figures and Tables.
(PDF)
