# Supplementary material for: The Date of Interbreeding between Neandertals and Modern Humans
Source: PLoS Genet. 2012 Oct 4;8(10):e1002947. doi: 10.1371/journal.pgen.1002947 (PMC3464203; doi:10.1371/journal.pgen.1002947)
Supplement: Text S1 — Supporting Text including Figures and Tables. (PDF) [file pgen.1002947.s001.pdf]

Supporting Information Text S1

List of Supplementary Figures

S1 The fraction of SNPs s where there is an excess of Neandertal derived alleles n over Denisova derived alleles d as a function of the derived allele frequency in Europeans. 7

S2 Estimates of t_{GF} as a function of true t_{GF} for RGF I: We plot the mean and $2\times$ standard error of the estimates of t_{GF} from 100 independent simulated datasets using ascertainment 0. The estimates track the true t_{GF} though the variance increases for more ancient gene flow events. 11

S3 Classes of demographic models : a) Recent gene flow but no ancient structure. RGF I has no bottleneck in E. RGF II has a bottleneck in E after gene flow while RGF VI has a bottleneck in E before gene flow. RGF IV and V have constant population sizes of $N_e = 5000$ and $N_e = 50000$ respectively. b) Ancient structure but no recent gene flow. AS I has a constant population size while AS II has a recent bottleneck in E. c) Neither ancient structure nor recent gene flow. NGF I has a constant population size while NGF II has a recent bottleneck in E. d),e) Ancient structure + Recent gene flow. HM IV consists of continuous migration in the $Y-E$ ancestor and the $Y-E-N$ ancestor while HM I consists of continuous migration only in the $Y-E$ ancestor. HM II consist of a single admixture event in the ancestor of E while HM III also models a small population size in one of the admixing populations. 12

S4 A graphical model for map error estimation. Each circle denotes a random variable. Shaded circles indicate random variables that are observed. Plates indicate replicas of the random variables with the number of replicas denoted in the the top-left (*e.g.*, there are $m-1$ copies of Z_i).α is the parameter that measures the precision of the map. $G_i, i \in [m-1]$ refers to the observed genetic distances across the ith interval in the genetic map. We impose an exponential prior on α. G_i and α parameterize the distribution over the true, but unobserved, genetic distance Z_i. Z_i is gamma distributed with shape parameter αg_i and rate parameter α. The genetic distance of interval Z_i is partitioned amongst $[n_i]$ finer intervals to obtain genetic distances $Z_{i,j}$ using a Dirichlet distribution parameterized by β and the physical distances of the finer intervals. Given $Z_{i,j}$, the number of crossovers $C_{i,j}$ within interval (i,j) is given by a Poisson distribution with mean parameter $RZ_{i,j}$ where R is the total number of meioses observed. These crossovers are then uniformly distributed amongst all the windows that overlap interval (i,j). A crossover is observed within a window l, $Y_l = 1$, only if one of the intervals spanned by this window is assigned a crossover. 19
S5 Estimates of t_{GF} as a function of true t_{GF} for Demography RGF I: We plot the mean and $2\times$ standard error of the estimates of t_{GF} from 100 independent simulated datasets using ascertainment 1. The estimates track the true t_{GF} though the variance increases for more ancient gene flow events. 23
S6 Impact of the ascertainment scheme on the estimates of t_{GF} as a function of true t_{GF} for Demography RGF I: We plot the mean and $2\times$ standard error of the estimates of t_{GF} from 100 independent simulated datasets using ascertainment 2. 24
S7 Estimates of t_{GF} as a function of true t_{GF} for RGF I when the SNPs were filtered to mimic the 1000 genomes SNP calling process: We plot the mean and $2\times$ standard error of the estimates of t_{GF} from 100 independent simulated datasets using ascertainment 0. The estimates track the true t_{GF} and are indistinguishable from estimates obtained on the unfiltered dataset as seen in Figure S2. 25

S 8 Comparison of the LD decay conditioned on Neandertal derived alleles and Neandertal ancestral alleles stratified by the derived allele frequency in CEU (left) and YRI (right): In each panel, we compared the decay of LD for pairs of SNPs ascertained in two ways. One set of SNPs were chosen so that Neandertal carried the derived allele and where the number of derived alleles observed in the 1000 genomes CEU individuals is a parameter x. The second set of SNPs were chosen so that Neandertal carried only ancestral alleles and where the number of derived alleles observed in 1000 genomes CEU is x. We varied x from 1 to 12 (corresponding to a derived allele frequency of at most 10%). For each value of x, we estimated the extent of the LD *i.e.*, the scale parameter of the fitted exponential curve. Standard errors were estimated using a weighted block jackknife. Errorbars denote $1.96\times$ the standard errors. The extent of LD decay shows a different pattern in CEU vs YRI. In YRI, the extent of LD is similar across the two ascertainments to the limits of resolution although the point estimates indicate that the LD tends to be greater at sites where Neandertal carries the ancestral allele (8 out of 12). In CEU, on the other hand, the extent of LD is significantly larger at sites where Neandertal carries the derived allele (the only exception consists of singleton sites). Thus, the scale of LD at these sites must be conveying information about the date of gene flow. 28

List of Supplementary Tables

S1 Estimates of the time of gene flow for different demographies and mutation rates. . 11
S2 Correlation coefficient between times of gene flow estimated using haplotype and genotype data vs the true time of gene flow. 13
S3 Estimates of time of gene flow as a function of the quality of the genetic map: Data was simulated under a hotspot model of recombination. The observed genetic map was obtained by perturbing the true genetic map at a 1 Mb scale and then interpolating based on the physical positions of the markers. Smaller values of a indicate larger perturbation. λ denotes the estimates obtained on the perturbed map. t_{GF} denotes the estimates obtained after correcting for the errors in the observed map. Results are reported for two demographic models. 18
S4 Estimates of the precision of two genetic maps 18
S5 Estimates of time of gene flow for different demographies. For the demographies that involve recent gene flow (RGF II, RGF III, RGF IV and RGF V), the true time of gene flow is 2000 generations. 24
S6 Estimated time of the gene flow from Neandertals into Europeans (CEU) and East Asians (CHB+JPT): λ refers to the uncorrected time in generations obtained as described in Section S1. t_{GF} refers to the time in generations obtained from λ by integrating out the uncertainty in the genetic map as described in Section S3. y_{GF} refers to the time in years obtained from λ by integrating out the uncertainty in the genetic map and the uncertainty in the number of years per generation (we are reporting the posterior mean and 95% Bayesian credible intervals for each of these parameters). Estimates of the time of gene flow were obtained for CEU using the Decode map and the CEU LD map. Estimates for CHB+JPT were obtained using the CHB+JPT LD map (we do not have a precise estimate of the uncertainty in this genetic map – hence, we report only λ). 27
S7 Estimated time of the gene flow from Neandertals into Europeans (CEU) under different ascertainment schemes: λ refers to the uncorrected time in generations obtained as described in Section S1. Ascertainment 1 is shown to have a downward bias in the presence of bottlenecks since the gene flow – this may reflect the lower estimates obtained here. The estimates using Ascertainment 2 closely match the estimates shown in Table S6. 27
S8 Estimate of the time of gene flow stratified by distance to nearest exon (each bin contain 20% of the 1000 genome SNPs): These estimates were obtained on CEU using the Decode map. The results indicate that our estimates are not particularly sensitive to the strength of directional selection, which has recently been shown to be a widespread force in the genome [17, 18]. 29

Contents

S1 Statistic for dating 6
- S1.1 Statistic . . . 6
- S1.2 Preparation of 1000 genomes data . . . 7

S2 Simulation Results 8
- S2.1 Recent gene flow . . . 8
- S2.2 Ancient structure . . . 9
- S2.3 No gene flow . . . 9
- S2.4 Hybrid Models . . . 10
- S2.5 Effect of the mutation rate . . . 10

S3 Correcting for uncertainties in the genetic map 14
- S3.1 Correction . . . 14
- S3.2 Estimating α . . . 14
- S3.3 Inference . . . 16
- S3.4 Simulations . . . 17
- S3.5 Results . . . 17

S4 Uncertainty in the date estimates 20

S5 Effect of ascertainment 21
- S5.1 Recent gene flow . . . 21
- S5.2 Ancient structure . . . 21
- S5.3 No gene flow . . . 21
- S5.4 Hybrid Models . . . 21
- S5.5 Effect of the mutation rate . . . 22
- S5.6 Application to 1000 genomes data . . . 22

S6 Effect of the 1000 genomes SNP calling 25

S7 Effect of the 1000 genomes imputation 25

S8 Results 27

A Exponential decay of the statistic 30

B Proof of Equation 3 in Section S3 32

S1 Statistic for dating

A number of methods have been proposed to infer the demographic history and thus the population divergence times of closely-related species using multi-locus genotype data (see [1] and references therein). In this work, we seek to directly estimate the quantity of interest, *i.e*, the time of gene flow, by devising a statistic that is robust to demographic history. Our statistic is based on the pattern of LD decay due to admixture that we observe in a target population. The use of LD decay to test for gene flow is not entirely new ([2, 3]). [2] devised an LD-based statistic to test the hypotheses of recent gene flow vs ancient shared variation. [3] devised a statistic that used the decay of LD to obtain dates of recent gene flow events. The main challenge in our work is the need to estimate extremely old gene flow dates (at least 10000 years BP) while dealing with the uncertainty in recombination rates.

S1.1 Statistic

Consider three populations YRI, CEU and $Neandertal$, which we denote (Y, E, N). We want to estimate the date of last exchange of genes between N and E. In our demographic model, ancestors of (Y, E) and N split t_{NH} generations ago and Y and E split t_{YE} generations ago. Assume that the gene flow event happened t_{GF} generations ago with a fraction f of individuals from N. We have SNP data from several individuals in E and Y as well as low-coverage sequence data for N.

1. Pick SNPs according to an ascertainment scheme discussed below.

2. For all pairs of sites $S(x) = \{(i, j)\}$ at genetic distance x, consider the statistic $\overline{D}(x) = \frac{\sum_{(i,j)\in S(x)} D(i,j)}{|S(x)|}$. Here $D(i, j)$ is the classic signed measure of LD that measures the excess rate of occurrence of derived alleles at two SNPs compared to the expectation if they were independent [4].

3. If there was admixture and if our ascertainment picks pairs of SNPs that arose in Neandertal and introgressed (*i.e.*, these SNPs were absent in E before gene flow), we expect $\overline{D}(x)$ to have an exponential decay with rate given by the time of the admixture because $\overline{D}(x)$ is a consistent estimator of the expected value of D at genetic distance x. We can show that, under a model where gene flow occurs at a time t_{GF} and the truly introgressed alleles evolve according to Wright-Fisher diffusion, this expected value has an exponential decay with rate given by t_{GF}. Importantly, changes in population size do not affect the rate of decay although imperfections of the ascertainment scheme will affect this rate (see Appendix A for details).

We pick SNPs that are derived in N (at least one of the reads that maps to the SNP carries the derived allele), are polymorphic in E and have a derived allele frequency in $E < 0.1$. This ascertainment enriches for SNPs that arose in the N lineage and introgressed into E (in addition to SNPs that are polymorphic in the NH ancestor and are segregating in the present-day population). We chose a cutoff of 0.10 based on an analysis that computes the excess of the number of sites where Neandertal carries the derived allele compared to the number of sites where Denisova carries the derived allele stratified by the derived allele frequency in European populations ($\frac{(n-d)}{s}$ where s is the total number of polymorphic SNPs in Europeans). Given that Denisova and Neandertal are sister groups, we expect these numbers to be equal in the absence of gene flow. The magnitude of this excess is an estimate of the fraction of Neandertal introgressed alleles. Below a derived allele

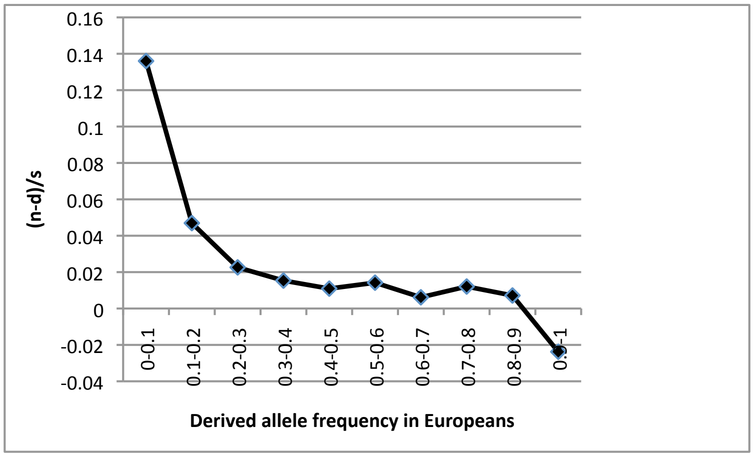

Figure S 1: The fraction of SNPs s where there is an excess of Neandertal derived alleles n over Denisova derived alleles d as a function of the derived allele frequency in Europeans.

frequency cutoff of 0.10 in Europeans, we see a significant enrichment of this statistic indicating that it is this part of the spectrum that is most informative for this analysis (see Figure S 1).

To further explore the properties of this ascertainment scheme, we performed coalescent simulations under the RGF II model discussed in Section S 2. We computed the fraction of ascertained SNPs for which the lineages leading to the derived alleles in E coalesce with the lineage in N before the split time of Neandertals and modern humans. This estimate provides us a lower bound on the number of SNPs that arose as mutations on the N lineage. We estimate that 30% of the ascertained SNPs arose as mutations in N leading to about 10-fold enrichment over the background rate of introgressed SNPs which has been estimated at $1 - 4\%$ [8].

We also explored other ascertainment schemes in Section S 5.

For the set of ascertained SNPs, we compute $\overline{D}(x)$ as a function of the genetic distance x and fit an exponential curve using ordinary least squares for x in the range of 0.02 cM to 1 cM in increments of 10^{-3} cM. The standard definition of D requires haplotype frequencies. To compute $D_{i,j}$ directly from genotype data, we estimated $D_{i,j}$ as the covariance between the genotypes observed at SNPs i and j [5]. We tested the validity of using genotype data on our simulations in Section S 2.

S1.2 Preparation of 1000 genomes data

We used the individual genotypes that were called as part of the pilot 1 of the 1000 genomes project [6] to estimate the LD decay. For each of the panels that were chosen as the target population in our analysis, we restricted ourselves to polymorphic SNPs. The SNPs were polarized relative to the chimpanzee base(PanTro2).

S2 Simulation Results

To test the robustness of our statistic, we performed coalescent-based simulations under demographic models that included recent gene flow, ancient structure and neither gene flow nor ancient structure. The classes of demographic models are shown in Figure S2.5

S2.1 Recent gene flow

S2.1.1 RGF I

In our first set of simulations, we generated 100 independent 1 Mb regions under a simple demographic model of gene flow from Neandertals into non-Africans. We set $t_{NH} = 10000$, $t_{YE} = 5000$. All effective population sizes are 10000. The fraction of gene flow was set to 0.03. We simulated 100 Y and E haplotypes respectively and 1 N haplotype. While we simulate a single haploid Neandertal, the sequenced Neandertal genome consists of DNA from 3 individuals. Hence, the reads obtained belong to one of 6 chromosomes. However, our statistic relies on the Neandertal genome sequence only to determine positions that carry a derived allele. We do not explicitly leverage any pattern of LD from this data. In our simulations, two SNPs at which Neandertal carries the derived allele necessarily lie on a single chromosome and ,hence, are more likely to be in LD than two similar SNPs in the sequenced Neandertals. However, the genetic divergence across the sequenced Vindija bones is quite low ([7] estimates the average genetic divergence to be about 6000 years) and so, we do not expect that this makes a big difference in practice.

We simulated 100 random datasets varying t_{GF} from 0 to 4500. Figure S5 shows the estimated t_{GF} tracks the true t_{GF} across the range of values of t_{GF}. As t_{GF} increases, the variance of our estimates increases – a result of the increasing influence of the non-admixture LD on the signals of ancient admixture LD. These results are encouraging given that our estimates were obtained using only about $\frac{1}{30}^{th}$ of the data that is available in practice. Further, to test the validity of the use of genotype data, we also computed Pearson's correlation r of estimates of t_{GF} obtained from genotype data to estimates obtained from haplotype data and we estimated these correlations to range from 0.89 to 0.96 across different true t_{GF} (see Table S2).

S2.1.2 RGF II

We assessed the effect of demographic changes since the gene flow on the estimates of the time of gene flow. We used $t_{NH} = 10000$, $t_{YE} = 2500$ and $t_{GF} = 2000$. The fraction of gene flow was set to 0.03. We simulated a bottleneck at 1020 generations of duration 20 generations in which the effective population size decreased to 100. We also simulated a 120 generation bottleneck in Neandertals from 3120 generations in which the effective population size decreased to 100. These parameters were chosen so that F_{st} between Y and E and the D-statistic $D(Y, E, N)$ match the observed values [8] (the value of the D-statistic $D(Y, E, N)$ depends on the probability of a European lineage entering the Neandertal population and coalescing with a Neandertal lineage before t_{NH} and could have been fit to the data by also adjusting f or t_{NH}) . We see in Table S1 that the estimated time remains unbiased.

S2.1.3 RGF III

We used a version of the demography used in [9] modified to match the F_{st} between Y and E and the D-statistics $D(Y, E, N)$. In this setup, $t_{NH} = 14400$, $t_{YE} = 2400$,$t_{GF} = 2000$, $f = 0.03$. Effective population sizes are 10000 in the E, YE ancestor, NH ancestor, and 10^6 in modern day Y. Modern day Y underwent exponential growth from a size of 10000 over the last 1000 generations. Y and E exchange genes after the split at a rate of 150 per generation. E underwent a bottleneck starting at 1440 generations that lasted 40 generations and had an effective population size of 320 during the bottleneck. We again generated 100 independent 1 Mb regions under this demography.

Table S1 shows that the estimates now have a small downward bias.

S2.1.4 RGF IV,V, VI

This is the same as RGF II but instead of a bottleneck we simulated a constant N_e in population E since gene flow. N_e was set to 5000 (RGF IV) and 50000 (RGF V). RGF VI places the bottleneck before the gene flow (the bottleneck begins at 2220 generations, has a duration of 20 generations in which the effective population size decreased to 100). Table S1 shows that the estimates remain accurate in these settings.

S2.2 Ancient structure

We examined if ancient structure could produce the signals that we see. We considered a demography (AS I) in which an ancestral panmictic population split to form the ancestors of modern-day Y and another ancestral population 15000 generations ago. The two populations had low-level gene flow (with population-scaled migration rate of 5 into Y and 2 leaving Y). The latter population split 9000 generations ago to form E and N. E and Y continued to exchange genes at a low-level down to the present (at a rate of 10). These parameters were again chosen to match the observed F_{st} between Y and E and $D(Y, E, N)$. Given the longer time scales (here and in the no gene flow model discussed next), we fit an exponential to our statistic over all distances up to 1 cM. We see from Table S1 that we estimate average times of around 10000 generations.

We also modified the above demography so that E experienced a 20 generation bottleneck that reduced their N_e to 100 that ended 1000 generations ago (AS II). Table S1 shows that our estimates are biased downwards significantly to around 5000 generations. Nevertheless, we also observe that the magnitude of the exponential, *i.e.*, its intercept, is also decreased. We also considered increasing the duration of the bottleneck but observed that the magnitude of the exponential decay is further diminished and becomes exceedingly noisy.

S2.3 No gene flow

We also considered a simple model of population splits without any gene flow from N to E (NGF I). We used $t_{NH} = 10000$, $t_{YE} = 2500$. To investigate if the observed decay of LD could be a result of variation in the effective population size, we also considered a variation (NGF II) with a bottleneck in E at 1020 generations of duration 20 generations in which the effective population size decreased to 100. Table S1 shows that our statistic estimates a date of around 8800 generations in NGF I which is reduced to around 5800 due to the bottleneck.

Our simulation results show that the LD-based statistic can accurately detect the timing of recent gene flow under a range of demographic models. On the other hand, population size changes in the target population can result in relatively recent dates when there is no gene flow or in the context of ancient structure. This motivated us to explore alternate ascertainment strategies in Section S5.

S2.4 Hybrid Models

These models consist of a recent gene flow from N to E but also simulate structure in the ancestral population of E *i.e.*, in E before gene flow. We would like to explore how ancestral structure affects estimates of the time of last gene exchange. In all these models, we set $t_{GF} = 2000, f = 0.03$. We consider several such models:

1. HM I: This is RGF II with no bottleneck in E. Instead, the ancestral population of E and Y is structured with the ancestors of E and Y exchanging migrants at a population-scaled rate of 5. This structure persists from $t_{NH} = 10000$ to $t_{YE} = 2500$ generations. The population ancestral to modern humans and Neandertal is panmictic.
2. HM II: Similar to HM I. The ancestral population of E is a $0.8 : 0.2$ admixture of two populations, E_1 and E_2, just prior to t_{GF}. E_1 split from Y at time t_{YE} while E_2 split from Y at time t_{NH} (resulting in a trifurcation at t_{NH}). .
3. HM III: Like in HM II, the ancestral population of E is admixed. E_2, in this model, has $N_e = 100$ throughout its history.
4. HM IV: This is similar to HM I. The structure in the ancestor of E and Y persists in the Neandertal-modern human ancestor. The ancestor now consists of two subpopulations exchanging migrants at a population-scaled rate of 5 till 15000 generations when the population becomes panmictic. N diverges from the subpopulation that is ancestral to E at time t_{NH}.

Table S 1 shows that t_{GF} is accurately estimated, albeit with a small upward bias, under these hybrid demographic models.

S2.5 Effect of the mutation rate

Mutation rate has an indirect effect on our estimates – the mutation rate affects the proportion of ascertained SNPs that are likely to be introgressed. We varied the mutation rate to 1×10^{-8} and 5×10^{-8} in the RGF II model with no European bottleneck and again obtained consistent estimates (Table S 1).

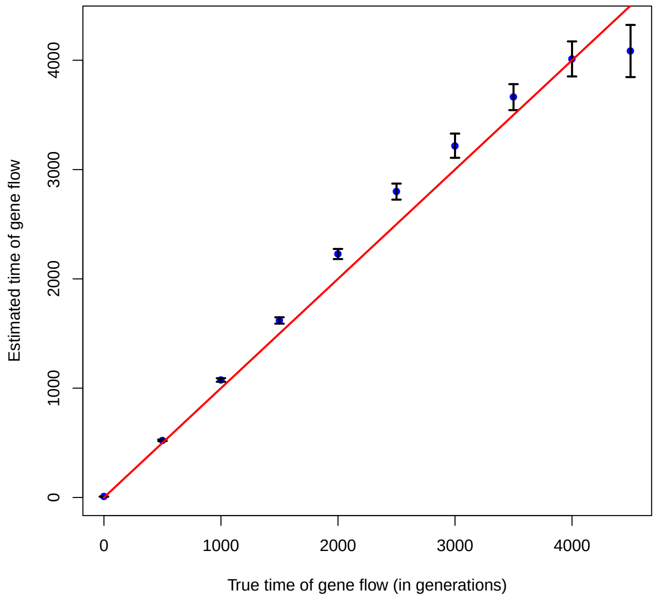

Figure S2: Estimates of t_{GF} as a function of true t_{GF} for RGF I: We plot the mean and $2\times$ standard error of the estimates of t_{GF} from 100 independent simulated datasets using ascertainment 0. The estimates track the true t_{GF} though the variance increases for more ancient gene flow events.

Demography	$F_{st}(Y,E)$	$D(Y,E,N)$	
RGF II	0.15	0.041	1987±48
RGF III	0.14	0.043	1776±87
RGF IV	0.15	0.04	2023 ± 56
RGF V	0.07	0.04	2157±22
RGF VI	0.15	0.04	2102 ± 36
AS I	0.15	0.045	10128±127
AS II	0.19	0.046	5070±397
NGF I	0.15	-21×10^{-5}	8847± 126
NGF II	0.15	9×10^{-5}	5800± 164
HM I	0.18	0.03	2174±40
HM II	0.12	0.04	2226±39
HM III	0.13	0.04	2137±34
HM IV	0.18	0.06	2153±36
Mutation rate	$F_{st}(Y,E)$	$D(Y,E,N)$	
1^{-8}	0.11	0.04	2141±41
5×10^{-8}	0.11	0.04	2134±41

Table S1: Estimates of the time of gene flow for different demographies and mutation rates.

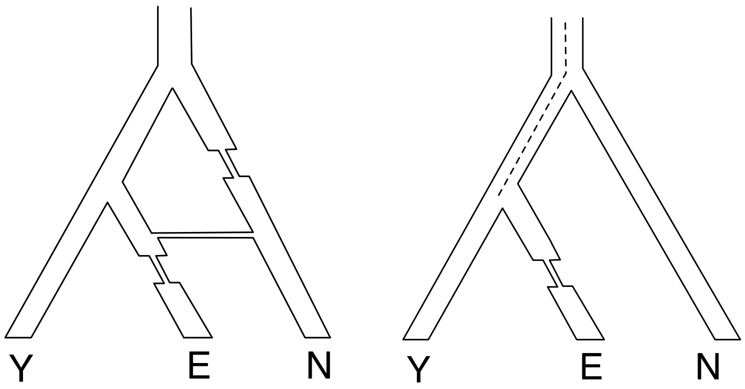

(a) RM: Recent gene flow (b) AS: Ancient structure

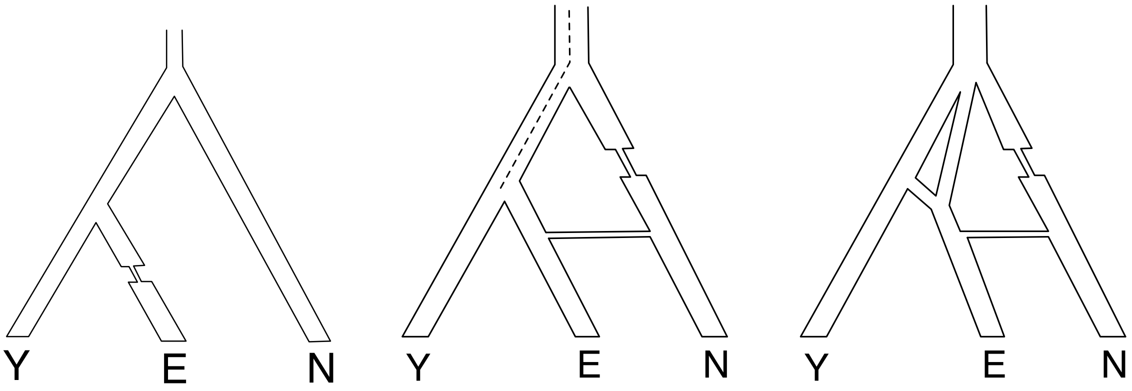

(c) NGF: No gene flow (d) HM: Hybrid model (e) HM: Hybrid model

Figure S3: Classes of demographic models : a) Recent gene flow but no ancient structure. RGF I has no bottleneck in E. RGF II has a bottleneck in E after gene flow while RGF VI has a bottleneck in E before gene flow. RGF IV and V have constant population sizes of $N_e = 5000$ and $N_e = 50000$ respectively. b) Ancient structure but no recent gene flow. AS I has a constant population size while AS II has a recent bottleneck in E. c) Neither ancient structure nor recent gene flow. NGF I has a constant population size while NGF II has a recent bottleneck in E. d),e) Ancient structure + Recent gene flow. HM IV consists of continuous migration in the $Y - E$ ancestor and the $Y - E - N$ ancestor while HM I consists of continuous migration only in the $Y - E$ ancestor. HM II consist of a single admixture event in the ancestor of E while HM III also models a small population size in one of the admixing populations.

True t_{GF}	Pearson's correlation
0	0.960918
500	0.9421455
1000	0.9335201
1500	0.9429699
2000	0.9339092
2500	0.9464859
3000	0.9378165
3500	0.8903148
4000	0.8884884
4500	0.9217262

Table S2: Correlation coefficient between times of gene flow estimated using haplotype and genotype data vs the true time of gene flow.

S3 Correcting for uncertainties in the genetic map

In this section, we show how uncertainties in the genetic lead to a bias in the estimates of the time of gene flow. We then show how we could correct our estimates assuming a model of map uncertainty. Our model characterizes the precision of a map by a single scalar parameter α. We estimate α for a given genetic map by comparing the distances between a pair of markers as estimated by the map to the number of crossovers that span those markers as observed in a pedigree. We propose a hierarchical model that relates α and the expected as well as observed number of crossovers and we infer an approximate posterior distribution of α by Gibbs sampling. Finally, we show using simulations that this procedure is effective in providing unbiased date estimates in the presence of map uncertainties and we apply this procedure to estimate the uncertainties of the Decode map and Oxford LD-based map by comparing these maps to crossover events observed in a Hutterite pedigree.

S3.1 Correction

We have a genetic map $\mathcal{G}$ defined on m markers. Each of the $m-1$ intervals is assigned a genetic distance $g_i, i \in \{1, \ldots, m-1\}$. These genetic distances provide a prior on the true underlying (unobserved) genetic distances Z_i. A reasonable prior on each Z_i is then given by

$$Z_i \sim \Gamma\left(\alpha g_i, \alpha\right) \tag{1}$$

where α is a parameter that is specific to the map. This implies that the true genetic distance Z_i has mean g_i and variance $\frac{g_i}{\alpha}$. So large values of α correspond to a more precise map. The above prior over Z_i has the important property that $Z_1 + Z_2 \sim \Gamma\left(\alpha(g_1 + g_2), \alpha\right)$ so that α is a property of the map and not of the specific markers used.

Given this prior on the true genetic distances, fitting an exponential curve to pairs of markers at a given observed genetic distance g, involves integrating over the exponential function evaluated at the true genetic distances given g *i.e.*,

$$\mathbb{E}\left[\exp\left(-t_{GF} Z\right) | g\right] = \exp\left(-\lambda g\right) \tag{2}$$

where λ is the rate of decay of $\overline{D}(g)$ as a function of the observed genetic distance g and can be estimated from the data in a straightforward manner and t_{GF} denotes the true time of the gene flow. It also easy to see that λ will be a downward biased estimate of t_{GF} (applying Jensen's inequality).

We can use Equation 1 to solve for t_{GF} (see Appendix B for details) as

$$t_{GF} = \alpha\left(\exp\left(\frac{\lambda}{\alpha}\right) - 1\right) \tag{3}$$

Thus, we need to estimate α for our genetic map to obtain an estimate of t_{GF}. As a check, note that for a highly precise map, $\alpha \gg \lambda$, we have $t_{GF} \approx \lambda$.

S3.2 Estimating α

Given a genetic map $\mathcal{G}$ defined on m markers, each of the $m-1$ intervals is assigned a genetic distance $g_i, i \in [m-1] = \{1, \ldots, m-1\}$. Each interval i may contain $n_i - 1, \geq 0$ additional markers not present in $\mathcal{G}$ that partition interval i into a finer grid of n_i intervals – each finer interval

is indexed by the set $T = \{(i,j), i \in [m-1], j \in [n_i]\}$ (*e.g.*, these additional markers could include markers that are found in the observed crossovers but not in the genetic map). Each interval (i,j) has a physical distance $p_{i,j}$.

We propose the following model for taking into account the effect of map uncertainty.

$$
\begin{aligned}
Z_i|\alpha, g_i &\sim \Gamma(\alpha g_i, \alpha) & (4)\\
(Z_{i,1},\ldots,Z_{i,n_i})|U_i, Z_i &\sim (U_{i,1}\ldots,U_{i,n_i})Z_i & (5)\\
U_i = (U_{i,1}\ldots,U_{i,n_i})|\beta &\sim \text{Dir}(\beta p_{i,1},\ldots,\beta p_{i,n_i}) & (6)
\end{aligned}
$$

The "true" genetic distance Z_i is related to the observed genetic distance g_i through the parameter α that is an estimate of map precision. The genetic distances of the finer intervals are obtained by partitioning the coarse intervals – the variability of this partition is controlled by the parameter β – β relates the physical distance to the genetic distance. When $\beta \to \infty$, the genetic distances of the finer grid are obtained by simply interpolating the coarse grid based on the physical distance.

Given the true genetic distances, we can now describe the probability of observing crossovers. Our observed data consists of R meioses that produce crossovers localized to L windows $\{I_1,\ldots,I_L\}$. Each window $l \in [L]$ consists of a set of contiguous intervals I_l and is known to contain a crossover event. Let $W_{i,j}$ denote the set of windows that overlap interval (i,j).

A note on our notation: $C_{i,j;l}$ is the number of crossovers in interval (i,j) that fall on window l. We can index the C variables by sets and then we are referring to the total number of crossovers in the index set *e.g.*, $C_{I_l;l}$ refers to all crossovers that fall on window l within the set of intervals I_l. Omitting an index from a random variable implies summing over that index. Thus, $C_{i,j} = \sum_{l=1}^{L} C_{i,j;l}$ denotes the number of crossover events in interval (i,j), $C_i = \sum_{j=1}^{n_i} C_{i,j}$ denotes the number of crossovers in the union of $(i,j), j \in [n_i]$. $\vec{\cdot}$ indicates a vector of random variables *e.g.*, $\vec{C}_S$ denotes the vector of counts indexed by the elements of set S.

If we assume that the probability of multiple crossovers in any of these intervals is small, we can use a simple probability model.

$$
\begin{aligned}
C_{i,j}|Z_{i,j} &\sim \text{Pois}(RZ_{i,j}) & (7)\\
\vec{C}_{i,j;l}|C_{i,j} &\propto \delta\left(\sum_{\{l\in W_{i,j}\}} C_{i,j;l} \leq C_{i,j}, C_{i,j;l} \in \{0,1\}, \sum_{\{l \notin W_{i,j}\}} C_{i,j;l} = 0\right) & (8)\\
Y_l|C_{i,j;l} &= \delta\left(C_{I_l} = \sum_{(i,j)\in I_l} C_{i,j;l} = 1\right) & (9)
\end{aligned}
$$

Here $C_{i,j}$ denotes the counts of crossover events within interval (i,j) over the R meioses and is a Poisson distribution with rate parameter $RZ_{i,j}$. In our model, $C_{i,j;l}$ is either zero or one and all the crossovers in interval (i,j) must fall on one of the $W_{i,j}$ windows that overlap (i,j). Finally, one of the $C_{i,j;l}$ within a window l must be one for a crossover to have been detected within this window ($Y_l = 1$).

We put an exponential prior on $\pi_\alpha \sim \exp(\frac{1}{\alpha_0})$ on α. We set $\alpha_0 = 10$ in our inference. While we can estimate β jointly, we instead fix β to ∞.

To summarize, the observations in our model consist of the $m-1$ observed genetic distances $G_i, i \in [m-1]$ and L observed crossovers from pedigree data $Y_l, l \in [L]$ (which often extend over multiple intervals in the underlying map) as well as the total number of meioses R in the pedigree.

The parameter of interest is α, a measure of the precision of the map. We impose an exponential prior on α. G_i and α parameterize the distribution over the true, but unobserved, genetic distance Z_i. Given the number of meioses and Z_i, the number of crossovers that fall within interval i (and is unobserved) is given by a Poisson distribution. These crossovers that fall within an interval i are then distributed uniformly at random amongst all the observed windows that overlap interval i. Finally, a crossover is observed only if one of the intervals spanned by it is assigned a crossover. Our model can also account for the fact that the genetic map has been estimated using only a subset of markers from a finer set of markers (so that the markers defining the map and those defining the crossover boundaries may be different): the genetic distance of interval Z_i is partitioned amongst the finer intervals $[n_i]$ to obtain genetic distances $Z_{i,j}$ using a Dirichlet distribution parameterized by β and the physical distances of the finer intervals; given these $Z_{i,j}$, we can again compute the probability of observing a crossover across these finer intervals.

Thus, we are interested in estimating the posterior probability $\pi(\alpha|\overrightarrow{Y}, \overrightarrow{G}, \beta)$ where $\overrightarrow{Y} = (Y_1, \ldots, Y_L)$, $\overrightarrow{G} = (G_1, \ldots, G_{m-1})$. $\pi(\alpha|\overrightarrow{Y}, \overrightarrow{G}, \beta) \propto \pi_\alpha(\alpha) \Pr(\overrightarrow{Y}|\alpha, \beta, \overrightarrow{G})$ where the likelihood is given by the probability model described above. To perform this inference, we set up a Gibbs sampler to estimate the posterior probability over the hidden variables $\pi(\alpha, \overrightarrow{Z}_{[m-1]}, \overrightarrow{U}_{[m-1]}, \overrightarrow{C}_T|\overrightarrow{Y}, \overrightarrow{G}, \beta)$.

S3.3 Inference

We perform Gibbs sampling to estimate the approximate posterior probability over the hidden variables $(\alpha, \overrightarrow{Z}_{[m-1]}, \overrightarrow{U}_{[m-1]}, \overrightarrow{C}_T)$. While a standard Gibbs sampler can be applied to this problem, mixing can be improved using the fact that we are interested in the estimates of α while the Z_i are nuisance parameters. We thus attempt to sample α given the $C_{i,j}$, integrating out the Z_i. We still need the Z_i in the model as it decouples the $C_{i,j}$. After sampling α, we resample the Z_i given the α and then resample $C_{i,j}$ given the resampled Z_i.

Given the parameter estimates at iteration $t-1$, their estimates at time t are given by

$$\begin{aligned}
\Pr(\alpha^{(t)}|\overrightarrow{C}_i^{(t-1)}) &\propto \prod_i \left(\frac{\Gamma\left(\alpha^{(t)} g_i + c_i\right)}{\Gamma\left(\alpha^{(t)} g_i\right)} \frac{{\alpha^{(t)}}^{\alpha^{(t)} g_i}}{(\alpha + R)^{c_i + \alpha^{(t)} g_i}} \right) \exp\left(-\frac{\alpha}{\alpha_0}\right) \\
Z_i^{(t)}|\alpha^{(t)}, C_i^{(t-1)} &\sim \Gamma\left(\alpha^{(t)} g_i + C_i^{(t-1)}, \alpha^{(t)} + R\right) \\
U_i^{(t)}|\beta, \overrightarrow{C}_{i,[n_i]}^{(t-1)} &\sim \text{Dir}\left(\overrightarrow{C}_{i,[n_i]}^{(t-1)} + \beta \overrightarrow{p}_{i,[n_i]}\right) \\
Z_{i,j}^{(t)}|U_i^{(t)}, Z_i^{(t)} &= U_{i,j}^{(t)} Z_i^{(t)}
\end{aligned}$$

In this sampler, $Z_{i,j}$ is a deterministic function of Z_i and C_i, so we can collapse $Z_{i,j}$.

The first equation samples α given the current estimates of the counts C_i. This is not a standard distribution. We sample from this distribution using an ARMS sampler [10].

The genetic distances between the markers in the original map $\overrightarrow{Z}_i$ is a gamma distribution with parameters updated by $C_i^{(t-1)}$. The genetic distances between the markers in the finer grid $Z_{i,j}$ can now be obtained by sampling the U_i which is a Dirichlet distribution with parameters updated by $C_i^{(t-1)}$.

We finally need to resample the counts $C_{i,j}$. For each window l, we can sample the total counts that fall within the window given the genetic distance spanned by the window (which in our simplified model is always 1 for each window). We then assign each of these counts to one of the intervals

within this window according to a multinomial distribution with probabilities proportional to their genetic distances. Finally $C_{i,j}$ is obtained by summing over the counts across all windows $W_{i,j}$ that overlap interval (i, j).

$$\begin{aligned}
\Pr(C^{(t)}_{I_l;l} | Y_l = 1, Z^{(t)}_{I_l}) &= \delta(C_{I_l;l} = 1) \\
C^{(t)}_{i,j;l} | C^{(t)}_{I_l;l}, Z^{(t)}_{I_l} &\sim Mult\left(1, Z^{(t)}_{I_l}\right) \\
C^{(t)}_{i,j} | C^{(t)}_{i,j;l} &= \sum_{l \in W_{i,j}} C^{(t)}_{i,j;l} \\
C^{(t)}_{i} | C^{(t)}_{i,j} &= \sum_{j=1}^{n_i} C^{(t)}_{i,j}
\end{aligned}$$

S3.4 Simulations

To investigate the adequacy of our model of map errors, we performed coalescent simulations using a hotspot model of recombination. We estimated the time of gene flow using an erroneous map. We then estimated the uncertainty of the parameter α by comparing the erroneous map to the true genetic map. We used the estimated α to obtain a corrected date. This procedure allows us to evaluate if our model can capture the uncertainties in the genetic map.

We simulated 100 independent 1 Mb regions using MSHOT [11]. We chose parameters for the recombination model similar to the parameters described in [12]. We considered a model with $t_{NH} = 10000, t_{YE} = 5000, t_{GF} = 2000$, constant effective population sizes of 10000 and a bottleneck in the Neandertal lineage of duration 200 generations and effective population size 100. Given the true genetic map for each locus, the observed map is a noisy version generated as follows: given the genetic map length l of each locus, the observed map has a genetic length G distributed according to a Gamma distribution $\Gamma(al, a)$ where a parameterizes the variance of the map [1]. Given G, the distances of the markers are obtained by interpolating from the physical positions.

We obtained an uncorrected estimate of the date λ using the observed genetic map. We then compared the true genetic map and the observed map to estimate α (restricting to markers at distances of at least 0.02 cM) and then obtained the corrected date t_{GF} according to Equation 3. Table S3 reports the results averaged over 10 random datasets. We see that the corrected date t_{GF} is quite accurate when the map is accurate at a scale of 1 Mb ($a \geq 1000$) and becomes less accurate when $a \leq 100$. The results are similar when we repeated the simulations with a demography in which there is a 20 generation bottleneck of $N_e = 100$ after the gene flow.

S3.5 Results

The previous results provide us confidence that the statistical correction for map uncertainty gives accurate estimates of the date provided the genetic map is reasonably accurate at a scale of 1 Mb. In our analyses, we therefore chose to use the Decode map [13] as well as the Oxford LD-based maps [14] which are known to be accurate at this scale. Another map that we considered using was

[1] Note that a is not the same as the parameter α that characterizes the variance of the true map given the observed map. a parameterizes the variance in an observed map given the true map while α parameterizes the variance in the true map given an observed map

a	No bottleneck since gene flow		Bottleneck	
	λ	t_{GF}	λ	t_{GF}
∞	1597±180	1926±252	1660 ±130	2005±194
1000	1653±198	2050±288	1715±127	2128±156
100	788±352	993±543	681±200	802±256

Table S 3: Estimates of time of gene flow as a function of the quality of the genetic map: Data was simulated under a hotspot model of recombination. The observed genetic map was obtained by perturbing the true genetic map at a 1 Mb scale and then interpolating based on the physical positions of the markers. Smaller values of a indicate larger perturbation. λ denotes the estimates obtained on the perturbed map. t_{GF} denotes the estimates obtained after correcting for the errors in the observed map. Results are reported for two demographic models.

a map obtained by using the physical positions to interpolate genetic distances estimated across entire chromosomes or sub-regions (e.g. the long arm, the centromere and the short arm). We chose not to use such a "physical" map because of its large variance at smaller size scales – *e.g.*, comparing this physical map to the Decode map suggests that the uncertainty in the genetic map is characterized by $a \approx 150$.

We estimated the uncertainty α of two maps – the Decode map and the CEU Oxford LD map. In each case, we assigned genetic distances to the SNPs in the 1000 genomes CEU data. Our observed crossovers consisted of the crossovers observed in a family of Hutterites [15]. We ran our Gibbs sampler for 500 iterations preceded by 250 iterations of burn-in (even though the mixing happens much faster). We initialized α from the prior. Different random initializations do not affect our results (even though this is not a diagnostic for problems with the chain or bugs). Our estimates show that the precision of the CEU LD map and the Decode map are quite similar with the Decode map being a little more accurate (see Table S4).

Map	α
Decode	1399.3±99.733
CEU	1221.89±78.79

Table S4: Estimates of the precision of two genetic maps

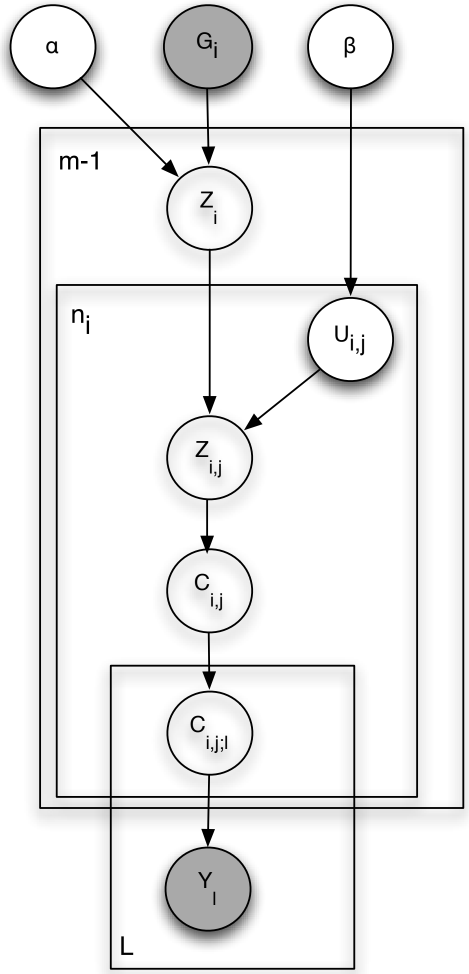

Figure S 4: A graphical model for map error estimation. Each circle denotes a random variable. Shaded circles indicate random variables that are observed. Plates indicate replicas of the random variables with the number of replicas denoted in the the top-left (*e.g.*, there are $m - 1$ copies of Z_i).α is the parameter that measures the precision of the map. $G_i, i \in [m-1]$ refers to the observed genetic distances across the ith interval in the genetic map. We impose an exponential prior on α. G_i and α parameterize the distribution over the true, but unobserved, genetic distance Z_i. Z_i is gamma distributed with shape parameter αg_i and rate parameter α. The genetic distance of interval Z_i is partitioned amongst $[n_i]$ finer intervals to obtain genetic distances $Z_{i,j}$ using a Dirichlet distribution parameterized by β and the physical distances of the finer intervals. Given $Z_{i,j}$, the number of crossovers $C_{i,j}$ within interval (i, j) is given by a Poisson distribution with mean parameter $RZ_{i,j}$ where R is the total number of meioses observed. These crossovers are then uniformly distributed amongst all the windows that overlap interval (i, j). A crossover is observed within a window l, $Y_l = 1$, only if one of the intervals spanned by this window is assigned a crossover.

S4 Uncertainty in the date estimates

We obtain estimates of the time of gene flow taking into account all sources of uncertainty. Denote the uncorrected date, the corrected date in generations and the corrected date in years by λ, t_{GF} and y_{GF} respectively.

Our model can be described as follows:

$$\begin{aligned} t_{GF} &= y_{GF} G \\ \lambda &= \alpha \left(\log \left(\frac{t_{GF}}{\alpha} \right) + 1 \right) \\ \overline{D}(x) &= a \exp\left(-\lambda x\right) + \epsilon \\ \epsilon &\sim N(0, \sigma^2) \\ \pi(\sigma^2) &\propto \frac{1}{\sigma^2} \end{aligned}$$

where $G \sim Unif(25, 33)$ denotes the number of years per generation, α is the uncertainty in the genetic map with prior given by the posterior estimated in Section S3 and $a \sim Unif(0, 1)$. Given this model, we can obtain the posterior probability distribution $\pi(\lambda|\overline{D}), \pi(t_{GF}|\overline{D}), \pi(y_{GF}|\overline{D})$ assuming a flat prior on each of the random variables λ, t_{GF}, y_{GF} respectively.

We obtain these posterior distributions by Gibbs sampling. We ran the Gibbs sampler for 200 burn-in iterations followed by 1000 iterations where we sampled every 10 iterations. We computed the posterior means and 95% credible intervals on λ, t_{GF} and y_{GF}.

S5 Effect of ascertainment

To test the robustness of our statistic, we performed coalescent-based simulations under the demographic models described in Section S 2. We explored two SNP ascertainments in addition to the ascertainment that we described in Section S 1 (which we refer to here as Ascertainment 0):

1. Ascertainment 1: SNPs for which Neandertal carries a derived allele, E is polymorphic and Y does not carry a derived allele.
2. Ascertainment 2: SNPs for which Neandertal carries a derived allele, E is polymorphic and Y does not carry a derived allele and SNPs for which Neandertal carries a derived allele, E does not carry a derived allele and Y is polymorphic.

S5.1 Recent gene flow

Under the simple demography I, Figures S 5 and S 6 show that, similar to ascertainment 0, the estimated t_{GF} tracks the true t_{GF} across the range of values of t_{GF} for ascertainments 1 and 2.

We assessed the effect of demographic changes since the gene flow on the estimates of the time of gene flow (demography RGF II of Section S 2). We see in Table S 5 that the bottleneck causes a downward bias in the estimated time using ascertainment 1 while ascertainment 2 is unbiased. For demography RGF III, Table S 5 shows that ascertainment 1 again has a downward bias on the estimated date while ascertainment 2 has a smaller upward bias.

S5.2 Ancient structure

In the AS I model, ascertainments 1 and 2 both produce estimate close to the time of last gene exchange (9000 generations) as does ascertainment 0. In AS II, however, both ascertainments are less affected by the recent bottleneck in population E and estimate older times that are closer to the true time of last gene exchange.

S5.3 No gene flow

Both ascertainments 1 and 2 produce dates that are quite old for both models NGF I and NGF II – the dates for NGF II are older than the estimates obtained using ascertainment 0. Ascertainment 2 produces estimates that are quite close to the time of last gene flow (t_{NH}).

Our simulation results show that in the case of recent gene flow, ascertainment 1 experiences a significant downward bias whereas ascertainment 2 is quite accurate. In the absence of gene flow or in the case of ancient structure, both ascertainments produce estimates that are quite old and they are more robust to population size changes in the target population relative to ascertainment 0.

S5.4 Hybrid Models

For all the hybrid models, we see that all the ascertainments are quite accurate with ascertainment 1 being most accurate while ascertainments 0 and 2 have a small upward bias.

S5.5 Effect of the mutation rate

Mutation rate has an indirect effect on our estimates – the mutation rate affects the proportion of ascertained SNPs that are likely to be introgressed. We varied the mutation rate to 1×10^{-8} and 5×10^{-8} in the RGF II model with no European bottleneck and again obtained consistent estimates (Table S5).

S5.6 Application to 1000 genomes data

Due to the process of SNP calling that calls SNPs separately in each population, SNPs called in one of the populations may not have calls in another. This is particularly problematic for SNPs that are polymorphic in one population and monomorphic in the other – precisely the SNPs that we would like to ascertain in the ascertainment schemes that we described above. To overcome this limitation, we used the following procedure to select our SNPs. For each of the SNPs that are polymorphic in the target population, we estimated the allele frequencies in the ancestral population directly from the reads that mapped to the SNP. We chose all SNPs whose derived allele frequency in the ancestral population is estimated to be less than 1% (since we have 118 YRI chromosomes, we can resolve frequencies of the order $\frac{1}{118} \approx 0.01$).

The ancestral allele, which was inferred using the Ensembl EPO alignment, was acquired from the 1000 Genomes Project FTP site. To derive the allele frequencies, we downloaded the pilot-phase alignments from the same FTP. We first adjusted each read alignment to avoid potential artifacts caused by short sequence insertions and deletions (INDELs), and then estimated the allele frequency by maximizing the likelihood using an estimation-maximization (EM) algorithm. More exactly, given we know the frequency $\phi^{(t)}$ at the t-th iteration, the estimate for the next round is:

$$\phi^{(t+1)} = \frac{1}{2n} \sum_{i=1}^{n} \frac{\sum_{g=0}^{2} g \mathcal{L}_i(g) f(g; 2, \phi^{(t)})}{\sum_{g=0}^{2} \mathcal{L}_i(g) f(g; 2, \phi^{(t)})}$$

where n is the total number of samples, $f(g; 2, \psi) = \binom{2}{g} \psi^g (1-\psi)^{2-g}$ is the frequency of genotype g under the Hardy-Weinberg equilibrium, and $\mathcal{L}_i(g)$ is the likelihood of g for the i-th sample. The genotype likelihood $\mathcal{L}_i(g)$ was computed using the MAQ error model [16].

The estimates of these different ascertainments are shown in Table S 5. We observe that, as in the simulations, the estimates obtained using ascertainment 1 are lower than the dates obtained using ascertainment 0 while those using ascertainment 2 are closer.

Finally, we also considered the effect of the frequency threshold of 0.10 used in Ascertainment 0. Using thresholds of 0.05 and 0.20, we obtain estimates of $\lambda = 1201(1172, 1233), 1188(1164, 1211)$ respectively using the Decode map. Thus, our estimates are not sensitive to the specific threshold chosen.

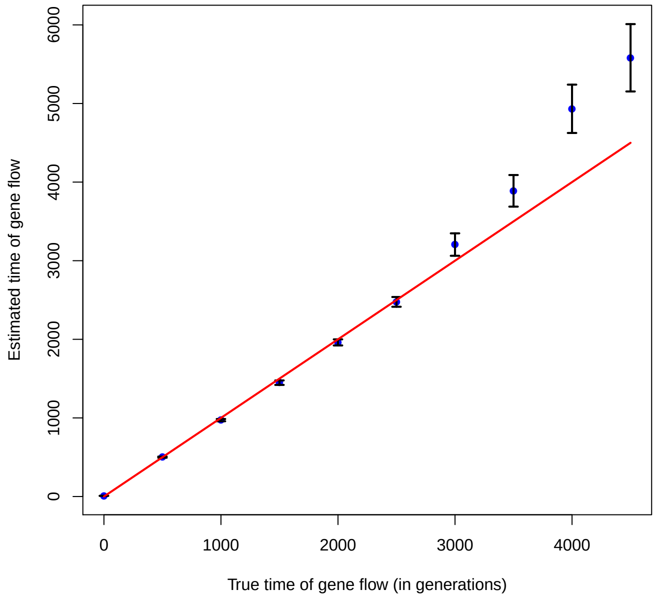

Figure S 5: Estimates of t_{GF} as a function of true t_{GF} for Demography RGF I: We plot the mean and $2\times$ standard error of the estimates of t_{GF} from 100 independent simulated datasets using ascertainment 1. The estimates track the true t_{GF} though the variance increases for more ancient gene flow events.

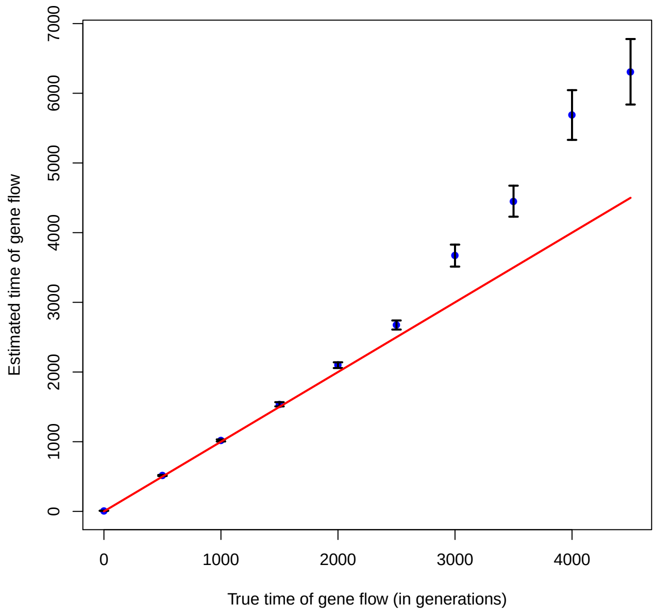

Figure S6: Impact of the ascertainment scheme on the estimates of t_{GF} as a function of true t_{GF} for Demography RGF I: We plot the mean and $2\times$ standard error of the estimates of t_{GF} from 100 independent simulated datasets using ascertainment 2.

Demography	Ascertainment 0	Ascertainment 1	Ascertainment 2
RGF II	1987±48	1693± 39	1960± 43
RGF III	1776±87	1642±98	2272±102
RGF IV	2023 ± 56	1751±36	1995 ± 38
RGF V	2157±22	2094 ± 22	2105 ± 22
RGF VI	2102±36	1814 ± 35	2029 ± 38
AS I	10128±127	8162±107	8861±110
AS II	5070±397	6349±327	7570±433
NGF I	8847±126	7940±257	10206±280
NGF II	5800± 164	7204± 356	11702± 451
HM I	2174±40	2057±36	2228±38
HM II	2226±39	2049±30	2100±30
HM III	2137±34	2040±29	2124±30
HM IV	2153±36	2038±34	2187±35
Mutation rate	Ascertainment 0	Ascertainment 1	Ascertainment 2
1^{-8}	2141±41	1847±35	1969±36
5×10^{-8}	2134±41	1833±29	1951±29

Table S5: Estimates of time of gene flow for different demographies. For the demographies that involve recent gene flow (RGF II, RGF III, RGF IV and RGF V), the true time of gene flow is 2000 generations.

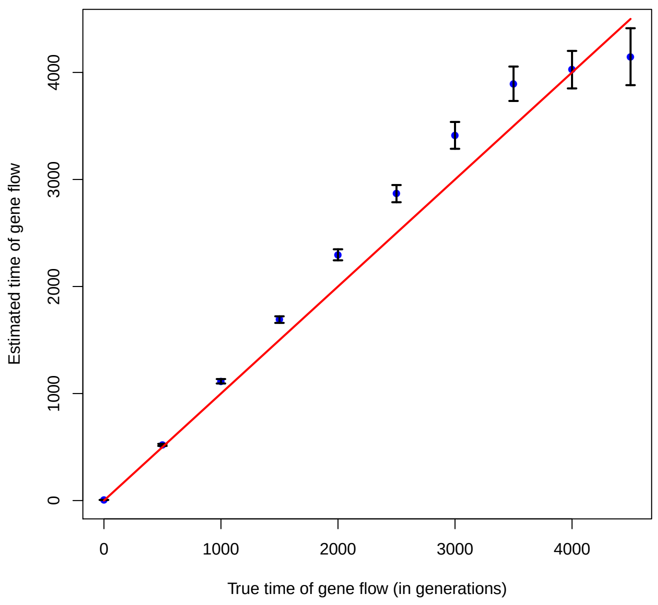

Figure S7: Estimates of t_{GF} as a function of true t_{GF} for RGF I when the SNPs were filtered to mimic the 1000 genomes SNP calling process: We plot the mean and $2\times$ standard error of the estimates of t_{GF} from 100 independent simulated datasets using ascertainment 0. The estimates track the true t_{GF} and are indistinguishable from estimates obtained on the unfiltered dataset as seen in Figure S2.

S6 Effect of the 1000 genomes SNP calling

One of the concerns with the estimates obtained from SNPs called in 1000 genomes arises from the low power to detect low-frequency alleles. To assess the effect of missing low-frequency variants on our inference, we redid the simulations in the RGF I model where SNPs were filtered to mimic the 1000 genomes SNP calling. Each SNP was retained in the dataset as a function of the number of copies of the minor allele – the acceptance probabilities are $0.25, 0.5, 0.75, 0.80, 0.9, 0.95, 0.96, 0.97, 0.98, 0.99$ for minor allele counts of $1, 2, 3, 4, 5, 6, 7, 8, \geq 9$ respectively. Figure S7 shows that the estimates on this filtered dataset are indistinguishable from the unfiltered dataset showing that the low power to call rare alleles does not affect our inference.

S7 Effect of the 1000 genomes imputation

A potential concern with interpreting our LD-based estimates applied to the SNPs called in 1000 genomes arises from the fact that genotype calling in the 1000 genomes project involves an imputation step which used LD in a reference panel to call genotypes [6]. It is unclear how this step affects our estimates. To understand the effect of imputation, we estimated the haplotype frequencies at pairs of SNPs directly from the 1000 genome reads aligned to the human reference hg18. We then used these haplotype frequencies to estimate LD (as opposed to the genotypic LD that we

use in the rest of the paper) [4].

Similar to the estimate of allele frequencies from the sequencing data, the two-locus haplotype frequencies are also estimated using an EM algorithm. Given k loci, let $\vec{h} = (h_1, \ldots, h_k)$ be a haplotype where h_j equals 1 if the allele at the j-th locus is derived, and equals 0 otherwise. Let $\eta_{\vec{h}}$ be the frequency of haplotype $\vec{h}$ satisfying $\sum_{\vec{h}} \eta_{\vec{h}} = 1$, where

$$\sum_{\vec{h}} = \sum_{h_1=0}^{1} \sum_{h_2=0}^{1} \cdots \sum_{h_k=0}^{1}$$

Knowing the genotype likelihood at the j-th locus for the i-th individual $\mathcal{L}_i^{(j)}(g)$, we can compute the haplotype frequencies iteratively with:

$$\eta_{\vec{h}}^{(t+1)} = \frac{\eta_{\vec{h}}^{(t)}}{n} \sum_{i=1}^{n} \frac{\sum_{\vec{h}'} \eta_{\vec{h}'}^{(t)} \prod_{j=1}^{k} \mathcal{L}_i^{(j)}(h_j + h'_j)}{\sum_{\vec{h}',\vec{h}''} \eta_{\vec{h}'}^{(t)} \eta_{\vec{h}''}^{(t)} \prod_j \mathcal{L}_i^{(j)}(h'_j + h''_j)} \tag{10}$$

We restricted our analysis to SNPs chosen using ascertainment 0 and used the Decode map to determine our genetic distances. We fitted an exponential with an affine term to the decay curve to obtain an uncorrected date $\lambda = 1210$, consistent with $\lambda = (1179, 1233)$ obtained using the genotypes called in 1000 genomes. Thus, the genotype imputation does not appear to be a major source of bias in our estimates.

S8 Results

Map	CEU			CHB+JPT		
	λ	t_{GF}	y_{GF}	λ	t_{GF}	y_{GF}
Decode	1201	1900	54540	–	–	–
	(1179,1233)	(1805,1993)	(47334,63146)	–	–	–
LD	1170	1961	56266	1269	–	–
	(1159,1183)	(1881,2043)	(49021,64926)	(1253,1287)	–	–

Table S6: Estimated time of the gene flow from Neandertals into Europeans (CEU) and East Asians (CHB+JPT): λ refers to the uncorrected time in generations obtained as described in Section S 1. t_{GF} refers to the time in generations obtained from λ by integrating out the uncertainty in the genetic map as described in Section S 3. y_{GF} refers to the time in years obtained from λ by integrating out the uncertainty in the genetic map and the uncertainty in the number of years per generation (we are reporting the posterior mean and 95% Bayesian credible intervals for each of these parameters). Estimates of the time of gene flow were obtained for CEU using the Decode map and the CEU LD map. Estimates for CHB+JPT were obtained using the CHB+JPT LD map (we do not have a precise estimate of the uncertainty in this genetic map – hence, we report only λ).

	Map	CEU		
		λ	t_{GF}	y_{GF}
Ascertainment 1	Decode	962	1385	39760
		(937,989)	(1328,1438)	(34593,45923)
	LD	1060	1694	48652
		(1045,1074)	(1633,1755)	(42386,56065)
Ascertainment 2	Decode	1105	1683	48311
		(1080,1136)	(1590,1779)	(41796,56092)
	LD	1128	1858	53332
		(1089,1170)	(1764,1952)	(46134,61982)

Table S7: Estimated time of the gene flow from Neandertals into Europeans (CEU) under different ascertainment schemes: λ refers to the uncorrected time in generations obtained as described in Section S 1. Ascertainment 1 is shown to have a downward bias in the presence of bottlenecks since the gene flow – this may reflect the lower estimates obtained here. The estimates using Ascertainment 2 closely match the estimates shown in Table S6.

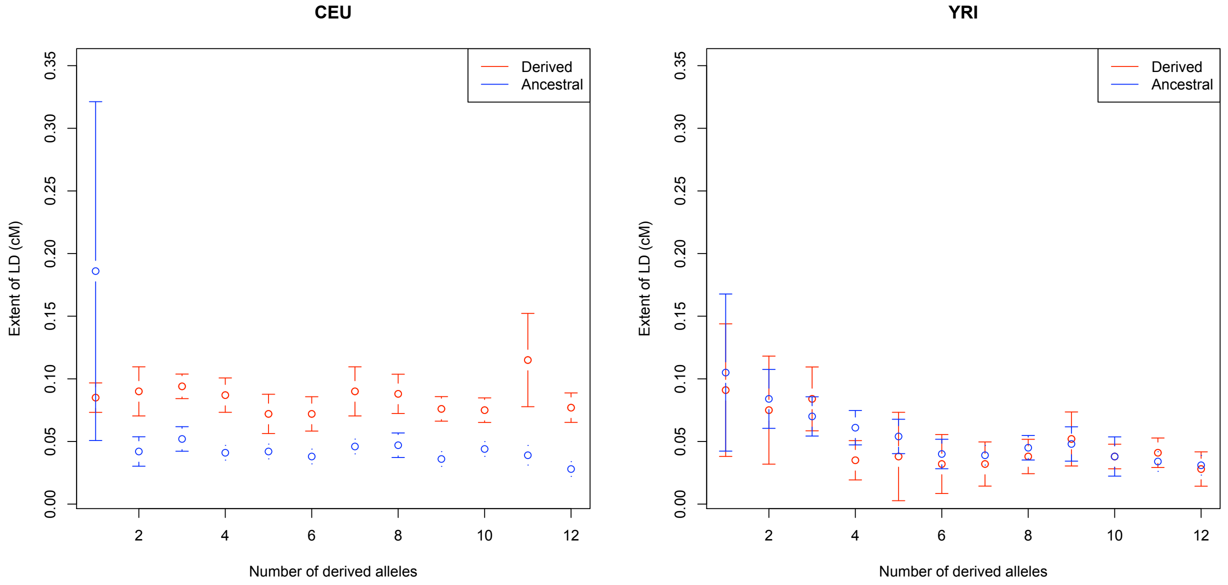

Figure S8: Comparison of the LD decay conditioned on Neandertal derived alleles and Neandertal ancestral alleles stratified by the derived allele frequency in CEU (left) and YRI (right): In each panel, we compared the decay of LD for pairs of SNPs ascertained in two ways. One set of SNPs were chosen so that Neandertal carried the derived allele and where the number of derived alleles observed in the 1000 genomes CEU individuals is a parameter x. The second set of SNPs were chosen so that Neandertal carried only ancestral alleles and where the number of derived alleles observed in 1000 genomes CEU is x. We varied x from 1 to 12 (corresponding to a derived allele frequency of at most 10%). For each value of x, we estimated the extent of the LD *i.e.*, the scale parameter of the fitted exponential curve. Standard errors were estimated using a weighted block jackknife. Errorbars denote $1.96\times$ the standard errors. The extent of LD decay shows a different pattern in CEU vs YRI. In YRI, the extent of LD is similar across the two ascertainments to the limits of resolution although the point estimates indicate that the LD tends to be greater at sites where Neandertal carries the ancestral allele (8 out of 12). In CEU, on the other hand, the extent of LD is significantly larger at sites where Neandertal carries the derived allele (the only exception consists of singleton sites). Thus, the scale of LD at these sites must be conveying information about the date of gene flow.

Distance to exon	λ	t_{GF}	y_{GF}
0-2475	1301	2149	61683
	(1256,1363)	(1991,2347)	(52737,72737)
2475-11028	1223	1967	56432
	(1176,1261)	(1874,2075)	(48708,65799)
11028-33707	1179	1847	53019
	(1131,1220)	(1717,1970)	(45679,61846)
33707-105107	1145	1773	50891
	(1098,1200)	(1640,1922)	(43330,59962)
105107-	1301	2151	61747
	(1253,1358)	(1982,2345)	(52442,73518)

Table S8: Estimate of the time of gene flow stratified by distance to nearest exon (each bin contain 20% of the 1000 genome SNPs): These estimates were obtained on CEU using the Decode map. The results indicate that our estimates are not particularly sensitive to the strength of directional selection, which has recently been shown to be a widespread force in the genome [17, 18].

A Exponential decay of the statistic

We are interested in how the linkage disequilibrium varies as a function of genetic distance x. We consider two SNPs that are polymorphic at time 0 in the past. The evolution of the alleles at the two SNPs can be described by the two-locus Wright-Fisher diffusion in a space parameterized by $X_t = (p, q, D)_t$ *i.e.*, the allele frequencies at each SNP at time t and measure of LD D at the two SNPs [19]. At time t, the average LD is denoted $\mathbb{E}D_t(x)$ (we assume that the population is not at equilibrium so that $\mathbb{E}D \neq 0$).

We are interested in the average linkage-disequilibrium at a time t given the state of the system at time $0 : u(t, x) = \mathbb{E}\left[D_t|X_0 = x\right]$.

We also denote the effective population size at time t by $N(t) = \nu(t)N_0$ and the probability of recombination between the two loci by r.

The evolution of $u(t, x)$ is given by

$$\frac{\partial u}{\partial t} = \mathcal{L}u \tag{11}$$

where $\mathcal{L}$ is the generator for this diffusion with initial condition

$$u(0, x) = D_0$$

and boundary conditions

$$\begin{aligned} u(t, (0, q, d)) = u(t, (p, 0, d) &= 0 \\ \frac{\partial u}{\partial d}(p, q, d_{max}(p, q)) = \frac{\partial y}{\partial d}(p, q, d_{min}(p, q)) &= 0 \end{aligned}$$

$$\frac{\partial u}{\partial t} = \mathcal{L}u = -\left[r + \frac{1}{2\nu(t)N_0}\right]u \tag{12}$$

The solution to Equation 12 is given by

$$u(t, x) \quad = \quad D_0 \exp\left(-\frac{1}{2N_0}\int_0^t \frac{d\tau}{\nu(\tau)}\right)\exp\left(-rt\right)$$

So we have

$$\mathbb{E}D_t \quad = \quad \mathbb{E}D_0 \exp\left(-\frac{1}{2N_0}\int_0^t \frac{d\tau}{\nu(\tau)}\right)\exp\left(-rt\right) \tag{13}$$

If we choose SNPs that that arose in the N lineage and introgressed into E t_{GF} generations ago (*i.e.*, these are SNPs that were monomorphic in E before the gene flow), Equation 13 says that the average D observed between all such pairs of SNPs at a given genetic distance r depends on three factors – the average LD at time 0 ($\mathbb{E}D_0$), the factor $\exp\left(-\frac{1}{2N_0}\int_0^t \frac{d\tau}{\nu(\tau)}\right)$ that accounts for changes in population sizes since gene flow and the factor $\exp\left(-rt\right)$ that accounts for the decay in LD. Terms 1 and 3 depend on r while term 2 does not. Further, since we ascertain SNPs that arose in the N lineage and introgressed into E, $\mathbb{E}D_0$ will depend on the average value of D in the introgressing Neandertals scaled by their admixing proportion. While $\mathbb{E}D_0$ still depends on the genetic distance r, for highly-bottlenecked populations such as the Neandertals ,in which the probability of coalescence has been estimated to be at least 0.65 [7], this term could be assumed

to be a constant in r. We can then approximate the relation between the average D and the genetic distance r by the exponential term $\exp(-rt_{GF})$ where the intercept of the exponential (its value at $r = 0$) depends on the population history. Thus, rate of decay of the expectation of D as a function of r would correspond in this case to t_{GF} and could provide a robust method to date gene flow.

Equation 13 implies that changes in the effective population size since the gene flow will not change the relation between $\mathbb{E}D_t$ and r. Since we have chosen SNPs that are monomorphic in E before the time of gene flow, demographic history in E before gene flow also does not affect $\mathbb{E}D_t$. However, this result has limitations when applied to polymorphism data. First, this result requires precisely ascertaining SNPs that arose in N and introgressed. Imperfections in the ascertainment can make the procedure sensitive to demography. Further, the expectation needs to be computed over all pairs of SNPs that were polymorphic at time 0 even if these SNPs may have fixed or gone extinct since. Such SNPs would be hard to ascertain using present-day genomes. Second, if the drift since gene flow is high or the level of gene flow is low, the intercept of the exponential curve decreases making it harder to estimate its rate of decay from data

B Proof of Equation 3 in Section S3

Equation 2 is given by

$$\mathbb{E}\left[\exp\left(-t_{GF}Z\right)|g\right] = \exp\left(-\lambda g\right) \tag{14}$$

where

$$Z \sim \Gamma\left(\alpha g, \alpha\right) \tag{15}$$

We can explicitly compute the LHS of 2

$$\begin{aligned}
\mathbb{E}\left[\exp\left(-t_{GF}Z\right)|g\right] &= \frac{\alpha^{\alpha g}}{\Gamma(\alpha g)} \int dz z^{\alpha g-1} \exp\left(-\alpha z\right) \exp\left(-t_{GF} z\right) \\
&= \frac{\alpha^{\alpha g}}{\Gamma(\alpha g)} \frac{\Gamma(\alpha g)}{\left(t_{GF}+\alpha\right)^{\alpha g}} \\
&= \exp\left(-\alpha \log\left(\left(\frac{t_{GF}}{\alpha}\right) + 1\right) g\right) \qquad (16)
\end{aligned}$$

Equating the coefficients of g in the RHS of Equation 2 and 16 gives us Equation 3.

References

[1] Catarina Pinho and Jody Hey. Divergence with gene flow: Models and data. *Annual Review of Ecology, Evolution, and Systematics*, 41(1):215–230, 2010.

[2] C. A. Machado, R. M. Kliman, J. A. Markert, and J. Hey. Inferring the history of speciation from multilocus DNA sequence data: the case of Drosophila pseudoobscura and close relatives. *Mol. Biol. Evol.*, 19:472–488, Apr 2002.

[3] Priya Moorjani, Nick Patterson, Joel N. Hirschhorn, Alon Keinan, Li Hao, Gil Atzmon, Edward Burns, Harry Ostrer, Alkes L. Price, and David Reich. The history of african gene flow into southern europeans, levantines, and jews. *PLoS Genet*, 7(4):e1001373, 04 2011.

[4] Lewontin Richard and Kojima Ken-Ichi. The evolutionary dynamics of complex polymorphisms. *Evolution*, 14:458 – 472, 1960.

[5] Weir Bruce. *Genetic Data Analysis III*. Sinauer Associates, 3rd edition, 2010.

[6] Richard M. Durbin and 1000 Genomes Project Consortium. A map of human genome variation from population-scale sequencing. *NATURE*, 467(7319):1061–1073, OCT 28 2010.

[7] D. Reich, R. E. Green, M. Kircher, J. Krause, N. Patterson, E. Y. Durand, B. Viola, A. W. Briggs, U. Stenzel, P. L. Johnson, T. Maricic, J. M. Good, T. Marques-Bonet, C. Alkan, Q. Fu, S. Mallick, H. Li, M. Meyer, E. E. Eichler, M. Stoneking, M. Richards, S. Talamo, M. V. Shunkov, A. P. Derevianko, J. J. Hublin, J. Kelso, M. Slatkin, and S. Paabo. Genetic history of an archaic hominin group from Denisova Cave in Siberia. *Nature*, 468:1053–1060, Dec 2010.

[8] Richard E. Green, Johannes Krause, Adrian W. Briggs, Tomislav Maricic, Udo Stenzel, Martin Kircher, Nick Patterson, Heng Li, Weiwei Zhai, Markus Hsi-Yang Fritz, Nancy F. Hansen, Eric Y. Durand, Anna-Sapfo Malaspinas, Jeffrey D. Jensen, Tomas Marques-Bonet, Can Alkan, Kay Prüfer, Matthias Meyer, Hernán A. Burbano, Jeffrey M. Good, Rigo Schultz, Ayinuer Aximu-Petri, Anne Butthof, Barbara Höber, Barbara Höffner, Madlen Siegemund, Antje Weihmann, Chad Nusbaum, Eric S. Lander, Carsten Russ, Nathaniel Novod, Jason Affourtit, Michael Egholm, Christine Verna, Pavao Rudan, Dejana Brajkovic, Željko Kucan, Ivan Gušic, Vladimir B. Doronichev, Liubov V. Golovanova, Carles Lalueza-Fox, Marco de la Rasilla, Javier Fortea, Antonio Rosas, Ralf W. Schmitz, Philip L. F. Johnson, Evan E. Eichler, Daniel Falush, Ewan Birney, James C. Mullikin, Montgomery Slatkin, Rasmus Nielsen, Janet Kelso, Michael Lachmann, David Reich, and Svante Pääbo. A draft sequence of the neandertal genome. *Science*, 328(5979):710–722, 2010.

[9] Jeffrey D. Wall, Kirk E. Lohmueller, and Vincent Plagnol. Detecting ancient admixture and estimating demographic parameters in multiple human populations. *Molecular Biology and Evolution*, 2009.

[10] W. R. Gilks, N. G. Best, and K. K. C. Tan. Adaptive rejection metropolis sampling. *Applied Statistics*, 44:455–472, 1995.

[11] Garrett Hellenthal and Matthew Stephens. mshot: modifying hudson's ms simulator to incorporate crossover and gene conversion hotspots. *Bioinformatics*, 23(4):520–521, 2007.

[12] Garrett Hellenthal, Adam Auton, and Daniel Falush. Inferring human colonization history using a copying model. *PLoS Genet*, 4(5):e1000078, 05 2008.

[13] A. Kong, G. Thorleifsson, D. F. Gudbjartsson, G. Masson, A. Sigurdsson, A. Jonasdottir, G. B. Walters, A. Jonasdottir, A. Gylfason, K. T. Kristinsson, S. A. Gudjonsson, M. L. Frigge, A. Helgason, U. Thorsteinsdottir, and K. Stefansson. Fine-scale recombination rate differences between sexes, populations and individuals. *Nature*, 467:1099–1103, Oct 2010.

[14] Simon Myers, Leonardo Bottolo, Colin Freeman, Gil McVean, and Peter Donnelly. A fine-scale map of recombination rates and hotspots across the human genome. *Science*, 310(5746):321–324, 2005.

[15] G. Coop, X. Wen, C. Ober, J. K. Pritchard, and M. Przeworski. High-resolution mapping of crossovers reveals extensive variation in fine-scale recombination patterns among humans. *Science*, 319:1395–1398, Mar 2008.

[16] H. Li, J. Ruan, and R. Durbin. Mapping short DNA sequencing reads and calling variants using mapping quality scores. *Genome Res.*, 18:1851–1858, Nov 2008.

[17] Graham McVicker, David Gordon, Colleen Davis, and Phil Green. Widespread genomic signatures of natural selection in hominid evolution. *PLoS Genet*, 5(5):e1000471, 05 2009.

[18] James J. Cai, J. Michael Macpherson, Guy Sella, and Dmitri A. Petrov. Pervasive hitchhiking at coding and regulatory sites in humans. *PLoS Genet*, 5(1):e1000336, 01 2009.

[19] T. Ohta and M. Kimura. Linkage disequilibrium at steady state determined by random genetic drift and recurrent mutation. *Genetics*, 63:229–238, Sep 1969.
